# Supplementary material for: Validation of a culturally adapted Swedish-language version of the Death Literacy Index
Source: PLoS One. 2023 Nov 30;18(11):e0295141. doi: 10.1371/journal.pone.0295141 (PMC10688853; doi:10.1371/journal.pone.0295141)
Supplement: S1 Appendix — (DOCX) [file pone.0295141.s001.docx]

## S1 Appendix. Original English-language Death Literacy Index, with scale and sub-scale headings

**1. Practical knowing**

1.1. Talking support

*PLEASE RATE HOW DIFFICULT OR EASY YOU WOULD FIND THE FOLLOWING TALKING SUPPORT (on a scale of 1-5 between Not at all able to Very able)*

1. Talk about death, dying or grieving to a close friend

2. Talk about death, dying or grieving to a child

3. Talk to a newly bereaved person about their loss

4. Talk to a GP about support at home or in their place of care for a dying person

1.2 Hands-on care

*PLEASE RATE HOW DIFFICULT OR EASY YOU WOULD FIND THE FOLLOWING HANDS-ON SUPPORT.* *Undertake the following care duties for the dying* (on a scale of 1-5 between *Not at all able* to *Very able*)

5. Feeding a person or assisting them to eat

6. Bathing a person

7. Lifting a person or assisting to transfer them

8. Administering injections

**2. Learning from experience**

*PLEASE RATE HOW MUCH EACH OF THE BELOW STATEMENTS SOUND LIKE YOU (on a scale of 1-5 between Very untrue of me to Very true of me).*

*My previous experience of grief, loss or other significant life events has*

9. Increased my emotional strength to help others with death and dying processes

10. Led me to re-evaluate what is important and not important in life

11. Developed my wisdom and understanding

12. Made me more compassionate toward myself

13. Provided me with skills and strategies when facing similar challenges in the future

**3. Existing knowledge**

*PLEASE RATE HOW MUCH EACH OF THE BELOW STATEMENTS SOUND LIKE YOU (on a scale of 1-5 between Strongly disagree to Strongly agree)*

14. I know the law regarding dying at home

15. I feel confident in knowing what documents you need to complete in planning for death

16. I know how to navigate the health care system to support a dying person to receive care

17. I know how to navigate funeral services and options

18. I know how to access palliative care in my area

19. I have sufficient understanding of illness trajectories to make informed decisions around medical treatments available and how that will shape quality of end of life

20. I know about the contribution the cemetery staff can make at end of life

**4. Community capacity**

4.1 Accessing help

*PLEASE RATE YOUR LEVEL OF AGREEMENT WITH THE FOLLOWING STATEMENTS (on a scale of 1-5 between Strongly disagree to Strongly agree).*

*If I were to provide end of life care for someone, I know people who could help me*

21. Access community support

22. Provide day to day care for the dying person

23. Access equipment required for care

24. Access culturally appropriate support

25. Access emotional support for myself

4.2 Support groups

*PLEASE RATE YOUR LEVEL OF AGREEMENT WITH THE FOLLOWING STATEMENTS (on a scale of 1-5 between Strongly disagree to Strongly agree).*

*There are support groups in my community for*

26. People with life threatening illnesses

27. People who are dying

28. Carers for people who are dying

29. People who are grieving
