# Supplementary material for: Validation of a culturally adapted Swedish-language version of the Death Literacy Index
Source: PLoS One. 2023 Nov 30;18(11):e0295141. doi: 10.1371/journal.pone.0295141 (PMC10688853; doi:10.1371/journal.pone.0295141)
Supplement: S2 Appendix — (DOCX) [file pone.0295141.s002.docx]

S2 Appendix. Online survey, comprising the Swedish Death Literacy Index items and sociodemographic questions (in English).

Note: The DLI-S items are presented here in Swedish, just as they were displayed to survey participants (English translations of DLI-S items are provided in table 3). The sociodemographic questions are presented in English but were shown in Swedish in the survey.

*Hur beredd skulle du vara att prata om följande?*

*Ange ditt svar på en skala mellan 1-5, där 1=inte alls beredd och 5=mycket beredd*

1. Att prata om att dö, döden eller sorg med en nära vän

2. Att prata om att dö, döden eller sorg med ett barn

3. Att prata med en sörjande person om hens förlust

4. Att prata med vårdpersonal om stöd till en person som kommer att dö i hemmet eller på den plats där hen får vård

*Hur beredd skulle du vara att göra följande?*

*Ange ditt svar på en skala mellan 1-5, där 1=inte alls beredd och 5=mycket beredd*

5. Att mata en person eller hjälpa hen att äta

6. Att hjälpa en person med att duscha eller att tvätta sig

7. Att lyfta en person eller hjälpa till då en person förflyttas

8. Att ge sprutor

*Hur stämmer följande för dig?*

*Ange ditt svar på en skala mellan 1-5, där 1=stämmer inte alls och 5=stämmer mycket väl*

**Tidigare erfarenheter av sorg, att förlora någon eller andra viktiga händelser i livet har...**

9. Gjort mig mer känslomässigt förberedd att ge stöd till andra i processer kring döden

10. Fått mig att fundera på vad som är viktigt och mindre viktigt i livet

11. Gjort mig mer klok och gett mig ny förståelse

12. Ökat min medkänsla för mig själv

13. Gett mig beredskap att klara av liknande utmaningar i framtiden

*Hur stämmer följande för dig?*

*Ange ditt svar på en skala mellan 1-5, där 1=inte alls och 5=mycket väl*

14. Jag känner till de regelsystem som rör dödsfall i hemmet

15. Jag känner till att det finns dokument som kan hjälpa en person att planera inför döden

16. Jag vet tillräckligt mycket om hur vård och omsorg fungerar för att kunna stödja en person att få vård i livets slut

17. Jag känner till processen inför begravning, vart jag kan vända mig och vilka val som finns

18. Jag känner till hur jag får tillgång till palliativ vård i området där jag bor

19. Jag vet tillräckligt för att kunna ta beslut om medicinska behandlingar och förstå hur de kan påverka livskvaliteten i livets slut

20. Jag känner till olika sätt som personal vid begravningsplatser kan vara till hjälp vid begravning

*Hur stämmer följande för dig?*

*Ange ditt svar på en skala mellan 1-5, där 1=inte alls och 5=mycket väl*

**Om jag skulle ta hand om en person i livets slut, så känner jag till var jag kan vända mig för:**

21. Att få stöd i området där jag bor, exempelvis från föreningar eller frivilligorganisationer

22. Att få hjälp med att ge en person daglig vård under livets sista tid

23. Att skaffa hjälpmedel som behövs för vård

24. Att få tillgång till stöd som passar personens kultur

25. Att få tillgång till eget känslomässigt stöd

*Hur stämmer följande för dig?*

*Ange ditt svar på en skala mellan 1-5, där 1=inte alls och 5=mycket väl*

**Jag känner till att det finns stödgrupper för:**

26. Personer som har en sjukdom som kan leda till döden

27. Personer som inte har långt kvar att leva

28. Personer som tar hand om någon som är döende

29. Personer som sörjer

Sociodemographic questions

1. **What is your age?***

…… years

1. **What gender are you?**

- Man
- Woman
- Non-binary
- Transgender
- Do not wish to answer

1. **What is your highest level of completed education?**

- No education or primary school not finished
- Primary education (e.g. primary school, elementary school)
- Lower secondary education (e.g. middle school, junior high school)
- Upper secondary education, general or vocational (e.g. gymnasium, high school)
- Post-secondary education (e.g. vocational certificate)
- Higher general or vocational education (e.g., higher education diploma)
- Higher education, bachelor’s level or equivalent
- Higher education, master’s level or equivalent
- Higher education, doctorate level or equivalent
- Other education

1. **What is your employment status? (Choose the alternative(s) that best describes your situation)** [multiple choice]

**Paid employment**

- Full time employment
- Part time employment
- Self-employed

**Non-paid employment**

- Student
- Military/civil service
- Actively seeking work
- Full time homemaker
- Retired/pensioned
- Leave of absence
- Parental leave
- Sick leave

1. **Do you currently work, or have you worked, in any of the following sectors?**

- Health care
- Social care
- No

1. **What is your post code/zip code?***
2. **What is your relationship status? (Please choose the alternative(s) that best describe(s) your situation)** [allow multiple choice]

- Single
- In a relationship but not co-habiting
- Married/co-habiting with partner
- Widowed
- Other (please specify)
- Do not wish to answer

1. **Do you have children?**

- Yes, I have children living in the household
- Yes, but they do not live in the household
- No

1. **Which country were you born in?**

[drop-down list of countries]

1. **Which country was your father born in?**

[drop-down list of countries]

1. **Which country was your mother born in?**

[drop-down list of countries]

1. **What language is spoken in your home?**

- Only Swedish
- Primarily Swedish
- Other language

1. **How would you describe your overall health?**

- Excellent
- Very Good
- Good
- Fair
- Poor
- Do not wish to answer

1. **Which of these statements comes closest to your beliefs?**

- There is a personal God
- There is some sort of spirit of life force
- I don’t really know what to think
- I don’t really think there is any sort of spirit God or life force

1. **Have you worked for pay with people who are at their end of life?**

- No
- Yes, currently
- Not currently but within the past 2 years
- Not currently but more than 2 years ago

1. **Have you worked as a volunteer with people who are at their end of life?**

- No
- Yes, currently
- Not currently but within the past 2 years
- Not currently but more than 2 years ago

1. **Have you worked for pay to support people through grief and loss?**

- No
- Yes, currently
- Not currently but within the past 2 years
- Not currently but more than 2 years ago

1. **Have you worked as a volunteer to support people through grief and loss?**

- No
- Yes, currently
- Not currently but within the past 2 years
- Not currently but more than 2 years ago

1. **Have you completed any formal training focused on the end of life, palliative care, death, dying and bereavement, as an adult?**

- No
- Yes, training encompassing less than 1 week
- Yes, training encompassing 1-10 weeks
- Yes, training encompassing more than 10 weeks

**Please specify what kind of training**:

1. **Have you ever had any of the following experiences? (please choose all options that apply to you)**

- Experience of a family member’s, close relative’s, or friend’s death
- Experience of a relative’s or an acquaintance’s death
- Experience of own life-threatening illness
- Experience of **supporting** a person with life-threatening illness
- Experience of **supporting** a person who has lost a family member or friend
- Experience of **caring for** a relative at the end of life
- Professional experience of **caring for** a person at the end of life
- Other experience related to the end of life, please specify………………………
- None
- For each alternative checked except for “None”: **When did you have this experience?**
- Within the last 2 years
- 2-5 years ago
- More than 5 years ago

1. **Have you attended an event about the end of life, death, dying, or bereavement?**

- Yes (please specify)
- No
- If “Yes”: **When did you have this experience?**
- Within the last 2 years
- 2-5 years ago
- More than 5 years ago
