# Supplementary material for: Validation of a culturally adapted Swedish-language version of the Death Literacy Index
Source: PLoS One. 2023 Nov 30;18(11):e0295141. doi: 10.1371/journal.pone.0295141 (PMC10688853; doi:10.1371/journal.pone.0295141)
Supplement: S3 Appendix — (DOCX) [file pone.0295141.s003.docx]

S3 Appendix. Instructions for expert panel review

**Assessment of translation quality**

The following items are translations from the English-language death literacy index, developed to measure the construct death literacy. Please read each translated and original item. Rate the quality of the translation with consideration to:

i) the **semantic equivalence**, or linguistic correlation, of the translated and original item, e.g. whether the meaning of the two are similar, with respect to idiomatic differences

ii) the **cultural equivalence** of the item in the [new country] context. The item content should be relevant across cultures and reflect the social structure and healthcare system of the society in question.

*Both assessments are made on a scale from 1 to 4.*

You have the possibility to comment and suggest changes for how to improve the items throughout the survey.

**[Translated item 1…29] [Original item 1…29]**

**1) Does the [Swedish] and English item ask the same thing?**

1 = the translated item is not an equivalent translation

2 = the translated item needs some revision to be an equivalent translation

3 = the translated item needs minor changes to be an equivalent translation

4 = the translated item is an equivalent translation

**2) Is the translated item culturally equivalent to the original item?**

1 = the translated item is not cross-culturally equivalent

2 = the translated item needs some revision to be a cross-culturally equivalent translation

3 = the translated item needs minor changes to be a cross-culturally equivalent translation

4 = the translated item is a cross-culturally equivalent translation

Do you have any suggestions for how to improve this translation?

**Assessment of item content**

The following items have been developed to measure death literacy [see definition above]. Please read each item and assess it according to their content validity in terms of relevance and clarity.

i) **item relevance** is assessed with regard to how relevant it is to measure the construct of death literacy and how representative it is of the content of the scale to which it belongs.

ii) **item clarity** is assessed in relation to item construction, wording, and ambiguity.

*Both assessments are made on a scale from 1 to 4.*

You have the possibility to comment and suggest changes for how to improve the items throughout the survey.

**[Translated item 1]**

**1) Is this item relevant, e.g. representative of the construct the instrument measures?**

1 = item is not relevant [to the latent variable measured]

2 = item need some revision to be relevant

3 = item needs minor revision to be relevant

4 = item is relevant

Comments:

If the item is not relevant, what are your suggestions for making it more relevant?

**2) Is this item clear in terms of e.g. wording, ambiguity, and at an appropriate level for the general public to read and understand?**

1 = item is not clear

2 = item needs some revision to be clear

3 = item needs minor revision to be clear

4 = item is clear

Comments:

If the item is not clear, what are your suggestions for making it clearer?

**[Translated item 2…29]**
