# Supplementary material for: Validation of a culturally adapted Swedish-language version of the Death Literacy Index
Source: PLoS One. 2023 Nov 30;18(11):e0295141. doi: 10.1371/journal.pone.0295141 (PMC10688853; doi:10.1371/journal.pone.0295141)
Supplement: S1 Table — Notes: DLI-S items 4 and 19, which were found to be problematic in terms of clarity, relevance, and/or language, are presented in Swedish and English. Item revision is marked in bold, with reasoning presented in English. (DOCX) [file pone.0295141.s004.docx]

S4 Table. Matrix with detailed examples of the revision process of two DLI-S items throughout instrument adaptation and validation.

|  | Initial translation | After expert review | After cognitive interviews | After literacy review |
| --- | --- | --- | --- | --- |
| Item 4 | *Prata med vårdpersonal om stöd i hemmet eller på annan plats där en döende person vårdas* | ***Att*** *prata med vårdpersonal om* ***stöd till en döende person i hemmet*** *eller den plats där* ***denne*** *vårdas* | *Att* ***samtala*** *med vårdpersonal om stöd till en döende person i hemmet eller den plats där denne vårdas* | *Att prata med vårdpersonal om* ***stöd till en person*** ***som kommer att dö*** *i hemmet eller på den plats där* ***hen får vård*** |
| Reasoning: | Original item: *Talk to a GP* (general practitioner) *about support at home or in their place of care for a dying person.*  While there is a Swedish equivalent term for GP (“husläkare”), they are rarely involved in palliative care. Instead, ‘care staff’ was chosen as it is a common term with a broader conceptual meaning. | Experts point out that care staff is not semantically equivalent to GP but that it is a term more suitable to the Swedish care system. It is also suggested that the Swedish wording sounded artificial. | Participants consider “prata” (“talk”) too generic, i.e. it could imply small talk or just a quick exchange. Instead, “samtala” (“discuss”) was suggested to imply a more in-depth conversation | The literacy consultant states that “prata” (“talk”) is a much more common that would make the item statement more accessible to people with lower literacy levels. |
| Item 19 | *Jag har tillräcklig förståelse kring olika sjukdomars förlopp för att kunna ta informerade beslut om medicinska behandlingar och hur de kan påverka livskvaliteten i livets slutskede* | *Jag* ***förstår*** *tillräckligt om olika sjukdomars förlopp för att kunna ta informerade beslut om* ***tillgängliga*** *medicinska behandlingar och förstå hur de kan påverka livskvaliteten i livets slutskede* | *Jag förstår tillräckligt om olika sjukdomars förlopp för att kunna ta informerade beslut om tillgängliga medicinska behandlingar och förstå hur de kan påverka livskvaliteten i livets slutskede* | *Jag* ***vet*** *tillräckligt för att kunna* ***ta beslut*** *om medicinska behandlingar och förstå hur de kan påverka livskvaliteten* ***i livets slut*** |
| Reasoning: | Original item: *I have sufficient understanding of illness trajectories to make informed decisions around medical treatments available and how that will shape quality of end of life*  It was decided to translate illness trajectories using more common and concrete words for the Swedish item to avoid using medical jargon. | Experts point out that the Swedish item does not clarify that it concerns medical treatments available, the same way the original item does, making it possible to interpret as any type of medical treatment (e.g. experimental ones). Item statement is made specific to available care. | The item is said to be hard to understand as it is long and contains several logical steps. It is also found difficult to rate, since it’s unclear to participants whether they should rate their knowledge, decision-making capacity, or both. Furthermore, the term “informed decision” seems ambiguous, particularly for people without professional care backgrounds. | The item was made shorter and more concise. Suggestions from the literacy consultant led to additional changes to make the language easier to understand. |

Notes: DLI-S items 4 and 19, which were found to be problematic in terms of clarity, relevance, and/or language, are presented in Swedish and English. Item revision is marked in bold, with reasoning presented in English.
