# Supplementary material for: Validation of a culturally adapted Swedish-language version of the Death Literacy Index
Source: PLoS One. 2023 Nov 30;18(11):e0295141. doi: 10.1371/journal.pone.0295141 (PMC10688853; doi:10.1371/journal.pone.0295141)
Supplement: S2 Table — Notes: *Positive rating = number of experts rating the item 3 or 4 on a 4-point scale. I-CVI calculated as (n of raters rating 3 or 4/total n of raters). Scale-level CVI was calculated using average proportion of I-CVI values. (DOCX) [file pone.0295141.s005.docx]

## S5 Table. Ratings of relevance and clarity and calculated overall content validity index (CVI).

| Item | Relevance *(positive rating*)* | Relevance total score *(max=40)* | Clarity *(positive rating*)* | Clarity total score *(max=40)* | Total *(max=80)* | I-CVI |
| --- | --- | --- | --- | --- | --- | --- |
| 1 | 10/10 | 38 | 10/10 | 37 | 75 | 0.937 |
| 2 | 10/10 | 36 | 10/10 | 37 | 73 | 0.912 |
| 3 | 10/10 | 38 | 10/10 | 36 | 74 | 0.925 |
| 4 | 9/10 | 36 | 9/10 | 34 | 70 | 0.875 |
| 5 | 9/10 | 37 | 9/10 | 35 | 72 | 0.900 |
| 6 | 9/10 | 36 | 10/10 | 38 | 74 | 0.925 |
| 7 | 10/10 | 38 | 10/10 | 37 | 75 | 0.937 |
| 8 | 10/10 | 38 | 10/10 | 39 | 77 | 0.962 |
| 9 | 10/10 | 39 | 10/10 | 38 | 77 | 0.962 |
| 10 | 10/10 | 39 | 10/10 | 38 | 77 | 0.962 |
| 11 | 10/10 | 39 | 10/10 | 37 | 76 | 0.950 |
| 12 | 8/10 | 36 | 8/10 | 35 | 71 | 0.887 |
| 13 | 9/10 | 38 | 9/10 | 36 | 74 | 0.925 |
| 14 | 9/10 | 36 | 9/10 | 35 | 71 | 0.887 |
| 15 | 9/10 | 35 | 9/10 | 36 | 71 | 0.887 |
| 16 | 9/10 | 37 | 8/10 | 36 | 73 | 0.912 |
| 17 | 10/10 | 39 | 10/10 | 36 | 75 | 0.937 |
| 18 | 10/10 | 39 | 10/10 | 38 | 77 | 0.962 |
| 19 | 9/10 | 38 | 9/10 | 37 | 75 | 0.937 |
| 20 | 8/10 | 32 | 9/10 | 35 | 67 | 0.837 |
| 21 | 9/10 | 38 | 9/10 | 35 | 73 | 0.912 |
| 22 | 8/10 | 36 | 9/10 | 35 | 71 | 0.887 |
| 23 | 9/10 | 37 | 9/10 | 35 | 72 | 0.900 |
| 24 | 10/10 | 38 | 10/10 | 36 | 74 | 0.925 |
| 25 | 10/10 | 38 | 10/10 | 36 | 74 | 0.925 |
| 26 | 10/10 | 40 | 10/10 | 39 | 79 | 0.987 |
| 27 | 9/10 | 37 | 10/10 | 39 | 76 | 0.950 |
| 28 | 10/10 | 39 | 10/10 | 38 | 77 | 0.962 |
| 29 | 10/10 | 40 | 10/10 | 39 | 79 | 0.987 |
|  |  |  | Scale-level CVI_Ave_ | | | 0.926 |

Notes: *Positive rating=number of experts rating the item 3 or 4 on a 4-point scale. I-CVI calculated as (*n* of raters rating 3 or 4/total *n* of raters). Scale-level CVI was calculated using average proportion of I-CVI values.
