# Supplementary material for: Validation of a culturally adapted Swedish-language version of the Death Literacy Index
Source: PLoS One. 2023 Nov 30;18(11):e0295141. doi: 10.1371/journal.pone.0295141 (PMC10688853; doi:10.1371/journal.pone.0295141)
Supplement: S3 Table — **. Correlation is significant at the 0.01 level (2-tailed). *. Correlation is significant at the 0.05 level (2-tailed). (PDF) [file pone.0295141.s006.pdf]

S6 Table. Inter-item correlation matrix of DLI-S items.

	1	2	3	4	5	6	7	8	9	10	11	12	13	14	15	16	17	18	19	20	21	22	23	24	25	26	27	28	29
Item 1	--																												
Item 2	.56**	--																											
Item 3	.53**	.47**	--																										
Item 4	.52**	.47**	.59**	--																									
Item 5	.31**	.26**	.30**	.42**	--																								
Item 6	.29**	.31**	.35**	.39**	.73**	--																							
Item 7	.18**	.20**	.15**	.31**	.56**	.56**	--																						
Item 8	.21**	.21**	.23**	.32**	.42**	.46**	.46**	--																					
Item 9	.45**	.37**	.56**	.45**	.34**	.34**	.22**	.24**	--																				
Item 10	.34**	.22**	.35**	.34**	.29**	.26**	.17**	.17**	.51**	--																			
Item 11	.35**	.27**	.42**	.38**	.26**	.28**	.18**	.16**	.63**	.69**	--																		
Item 12	.27**	.18**	.26**	.29**	.17**	.17**	.16**	.13**	.42**	.53**	.62**	--																	
Item 13	.44**	.35**	.45**	.46**	.29**	.31**	.20**	.23**	.63**	.50**	.59**	.55**	--																
Item 14	.21**	.22**	.32**	.36**	.24**	.29**	.14**	.33**	.32**	.24**	.27**	.24**	.35**	--															
Item 15	.25**	.23**	.27**	.28**	.18**	.23**	.17**	.15**	.23**	.11*	.17**	.15**	.30**	.44**	--														
Item 16	.26**	.23**	.34**	.43**	.35**	.45**	.28**	.41**	.35**	.26**	.28**	.24**	.41**	.62**	.47**	--													
Item 17	.27**	.24**	.41**	.41**	.20**	.28**	.11*	.17**	.34**	.23**	.25**	.19**	.37**	.44**	.43**	.49**	--												
Item 18	.16**	.19**	.28**	.35**	.23**	.32**	.16**	.30**	.30**	.21**	.23**	.21**	.30**	.61**	.39**	.69**	.46**	--											
Item 19	.24**	.25**	.32**	.41**	.30**	.33**	.25**	.46**	.32**	.20**	.21**	.18**	.36**	.57**	.39**	.72**	.50**	.66**	--										
Item 20	.21**	.24**	.39**	.42**	.24**	.27**	.13**	.24**	.37**	.23**	.26**	.23**	.38**	.52**	.36**	.54**	.71**	.57**	.61**	--									
Item 21	.15**	.20**	.29**	.33**	.26**	.29**	.19**	.28**	.33**	.17**	.23**	.16**	.30**	.56**	.34**	.56**	.42**	.65**	.55**	.53**	--								
Item 22	.15**	.13**	.28**	.34**	.36**	.42**	.25**	.29**	.34**	.27**	.27**	.22**	.36**	.54**	.34**	.69**	.45**	.71**	.58**	.53**	.68**	--							
Item 23	.15**	.15**	.25**	.32**	.33**	.39**	.23**	.26**	.30**	.20**	.25**	.13**	.30**	.51**	.35**	.64**	.47**	.65**	.54**	.50**	.67**	.82**	--						
Item 24	.09*	.10*	.22**	.28**	.26**	.32**	.23**	.25**	.32**	.17**	.22**	.15**	.25**	.49**	.29**	.53**	.38**	.53**	.48**	.48**	.66**	.69**	.66**	--					
Item 25	.19**	.21**	.29**	.36**	.29**	.31**	.24**	.23**	.39**	.26**	.31**	.23**	.38**	.52**	.32**	.58**	.42**	.61**	.54**	.51**	.72**	.70**	.68**	.71**	--				
Item 26	.24**	.21**	.25**	.34**	.32**	.34**	.26**	.37**	.28**	.23**	.25**	.23**	.34**	.44**	.35**	.50**	.32**	.45**	.47**	.35**	.47**	.50**	.47**	.45**	.50**	--			
Item 27	.24**	.22**	.24**	.35**	.32**	.33**	.25**	.34**	.30**	.23**	.27**	.23**	.33**	.50**	.33**	.52**	.35**	.51**	.49**	.41**	.59**	.56**	.56**	.54**	.61**	.84**	--		
Item 28	.26**	.23**	.31**	.39**	.28**	.32**	.22**	.33**	.36**	.28**	.28**	.25**	.38**	.50**	.34**	.54**	.37**	.56**	.54**	.45**	.57**	.59**	.55**	.54**	.60**	.74**	.81**	--	
Item 29	.28**	.23**	.27**	.34**	.26**	.30**	.21**	.32**	.29**	.24**	.27**	.21**	.34**	.47**	.31**	.46**	.36**	.44**	.43**	.43**	.52**	.50**	.48**	.42**	.56**	.71**	.70**	.68**	--

**. Correlation is significant at the 0.01 level (2-tailed).

*. Correlation is significant at the 0.05 level (2-tailed).
