# Supplementary material for: Validation of a culturally adapted Swedish-language version of the Death Literacy Index
Source: PLoS One. 2023 Nov 30;18(11):e0295141. doi: 10.1371/journal.pone.0295141 (PMC10688853; doi:10.1371/journal.pone.0295141)
Supplement: Attachment — Submitted filename: PONE-D-22-01686_reviewer.pdf [file pone.0295141.s007.pdf]

PLOS ONE

Validation of a culturally adapted Swedish-language version of the Death Literacy Index

--Manuscript Draft--

**Manuscript Number:**	PONE-D-22-01686
**Article Type:**	Research Article
**Full Title:**	Validation of a culturally adapted Swedish-language version of the Death Literacy Index
**Short Title:**	Validation of the Swedish-language version of the Death Literacy Index
**Corresponding Author:**	Therese Johansson, MSc.<br>Karolinska Institutet<br>Huddinge, Stockholm SWEDEN
**Keywords:**	Instrument development; public health; Questionnaire Surveys; Reliability and Validity; Death education; end-of-life care
**Abstract:**	The death literacy index (DLI) was developed in Australia to measure death literacy, a set of experience-based knowledge needed to understand and act on end-of-life (EOL) care options but has not yet been validated outside its original context. The aim of this study was to develop a culturally adapted Swedish-language version of the DLI, the DLI-S, and assess sources of evidence for its validity in a Swedish context.<br>The study involved a multi-step process of translation and cultural adaptation and two validation phases: examining first content and response process validity through expert review (n =10) and cognitive interviews (n =10); and second, internal structure validity of DLI-S data collected from an online cross-sectional survey (n =503). The psychometric evaluation involved analysis of descriptive statistics on item and scale-level, internal consistency and test-retest reliability, and confirmatory factor analysis.<br>During translation and adaptation, changes were made to adjust items to the Swedish context. Additional adjustments were made following findings from the expert review and cognitive interviews. The content validity index exceeded recommended thresholds. The psychometric evaluation provided support for DLI-S' validity. The hypothesized six-factor model showed good fit. High internal consistency reliability was demonstrated for the overall scale (Cronbach's $\alpha = 0.94$) and each sub-scale (α 0.81–0.93). Test-retest reliability was acceptable, ICC ranging between 0.66–0.85.<br>Through a comprehensive assessment of several sources of evidence, we show that the DLI-S demonstrates satisfactory validity and acceptability to measure death literacy in the Swedish context. There are, however, indications that the sub-scales measuring community capacity perform worse in comparison to other scales and may function differently in Sweden than in the original context. The DLI-S has potential to contribute to research on community-based EOL interventions.
**Order of Authors:**	Therese Johansson, MSc.
	Åsa Olsson, MSc., RN
	Carol Tishelman, PhD, RN
	Kerrie Noonan, BA(Psych), MPsych(Clinical), PhD
	Rosemary Leonard, PhD, BA
	Lars E. Eriksson, PhD, RN
	Ida Goliath, PhD, RN
	Joachim Cohen, PhD, MSc.
**Additional Information:**	
**Question**	**Response**
**Financial Disclosure**	This study was funded by Strategic Research Area Health Care Science (SFO-V), Karolinska Institutet; the Doctoral School in Health Care Sciences, Karolinska Institutet;

Powered by Editorial Manager® and ProduXion Manager® from Aries Systems Corporation

Enter a financial disclosure statement that describes the sources of funding for the work included in this submission. Review the submission guidelines for detailed requirements. View published research articles from *PLOS ONE* for specific examples.<br><br>This statement is required for submission and **will appear in the published article** if the submission is accepted. Please make sure it is accurate.<br><br>**Unfunded studies**<br>Enter: *The author(s) received no specific funding for this work.*<br><br>**Funded studies**<br>Enter a statement with the following details:<br>• Initials of the authors who received each award<br>• Grant numbers awarded to each author<br>• The full name of each funder<br>• URL of each funder website<br>• Did the sponsors or funders play any role in the study design, data collection and analysis, decision to publish, or preparation of the manuscript?<br>• **NO** - Include this sentence at the end of your statement: *The funders had no role in study design, data collection and analysis, decision to publish, or preparation of the manuscript.*<br>• **YES** - Specify the role(s) played.<br><br>* typeset	Swedish Research Council for Health, Welfare and Working Life (FORTE); Stockholm City Elder Care Bureau; and Stockholm Gerontology Research Center. The funders had no part in, nor influence on, the study design, data collection, analysis, interpretation, or writing of results.
**Competing Interests**<br><br>Use the instructions below to enter a competing interest statement for this submission. On behalf of all authors, disclose any competing interests that could be perceived to bias this work—acknowledging all financial support and any other relevant financial or non-financial competing interests.<br><br>This statement is required for submission and **will appear in the published article** if the submission is accepted. Please make	The authors have declared that no competing interests exist.

sure it is accurate and that any funding sources listed in your Funding Information later in the submission form are also declared in your Financial Disclosure statement.<br><br>View published research articles from *PLOS ONE* for specific examples.<br><br>**NO authors have competing interests**<br><br>Enter: *The authors have declared that no competing interests exist.*<br><br>**Authors with competing interests**<br><br>Enter competing interest details beginning with this statement:<br><br>*I have read the journal's policy and the authors of this manuscript have the following competing interests: [insert competing interests here]*<br><br>* typeset	
**Ethics Statement**<br><br>Enter an ethics statement for this submission. This statement is required if the study involved:<br><br>• Human participants<br>• Human specimens or tissue<br>• Vertebrate animals or cephalopods<br>• Vertebrate embryos or tissues<br>• Field research<br><br>Write "N/A" if the submission does not require an ethics statement.<br><br>General guidance is provided below. Consult the submission guidelines for detailed instructions. **Make sure that all information entered here is included in the Methods section of the manuscript.**	The study was approved by the Swedish Ethics Review Authority (reference number 2021-00915). All participants provided written informed consent, except participants in the cognitive interviews who provided oral informed consent.

Powered by Editorial Manager® and ProduXion Manager® from Aries Systems Corporation

**Format for specific study types**<br><br>**Human Subject Research (involving human participants and/or tissue)**<br>• Give the name of the institutional review board or ethics committee that approved the study<br>• Include the approval number and/or a statement indicating approval of this research<br>• Indicate the form of consent obtained (written/oral) or the reason that consent was not obtained (e.g. the data were analyzed anonymously)<br><br>**Animal Research (involving vertebrate animals, embryos or tissues)**<br>• Provide the name of the Institutional Animal Care and Use Committee (IACUC) or other relevant ethics board that reviewed the study protocol, and indicate whether they approved this research or granted a formal waiver of ethical approval<br>• Include an approval number if one was obtained<br>• If the study involved *non-human primates*, add *additional details* about animal welfare and steps taken to ameliorate suffering<br>• If anesthesia, euthanasia, or any kind of animal sacrifice is part of the study, include briefly which substances and/or methods were applied<br><br>**Field Research**<br><br>Include the following details if this study involves the collection of plant, animal, or other materials from a natural setting:<br>• Field permit number<br>• Name of the institution or relevant body that granted permission	
**Data Availability**<br><br>Authors are required to make all data underlying the findings described fully available, without restriction, and from the time of publication. PLOS allows rare exceptions to address legal and ethical concerns. See the PLOS Data Policy and FAQ for detailed information.	No - some restrictions will apply

Powered by Editorial Manager® and ProduXion Manager® from Aries Systems Corporation

A Data Availability Statement describing where the data can be found is required at submission. Your answers to this question constitute the Data Availability Statement and **will be published in the article**, if accepted.<br><br>**Important:** Stating 'data available on request from the author' is not sufficient. If your data are only available upon request, select 'No' for the first question and explain your exceptional situation in the text box.<br><br>Do the authors confirm that all data underlying the findings described in their manuscript are fully available without restriction?	
**Describe where the data may be found in full sentences. If you are copying our sample text, replace any instances of XXX with the appropriate details.**<br><br>• If the data are **held or will be held in a public repository**, include URLs, accession numbers or DOIs. If this information will only be available after acceptance, indicate this by ticking the box below. For example: *All XXX files are available from the XXX database (accession number(s) XXX, XXX.).*<br>• If the data are all contained **within the manuscript and/or Supporting Information files**, enter the following: *All relevant data are within the manuscript and its Supporting Information files.*<br>• If neither of these applies but you are able to provide **details of access elsewhere**, with or without limitations, please do so. For example:<br><br>*Data cannot be shared publicly because of [XXX]. Data are available from the XXX Institutional Data Access / Ethics Committee (contact via XXX) for researchers who meet the criteria for access to confidential data.*<br><br>*The data underlying the results presented in the study are available from (include the name of the third party*	Data cannot be deposited in a public repository because informed consent to deposit individual data was not obtained. Data are available on request from: Head of archives, Department of Neurobiology, Care Sciences and Society, Alfred Nobels Allé 23, Karolinska Institutet, SE-141 83 Huddinge, Sweden.

and contact information or URL).<br>• This text is appropriate if the data are owned by a third party and authors do not have permission to share the data.<br><br>* typeset	
Additional data availability information:	

Manuscript DLI-S validation
Click here to access/download;Manuscript;DLI-S validation_Final manuscript for submission.docx

Validation of a culturally adapted Swedish-language version of the Death Literacy Index

Therese Johansson[1]*, Åsa Olsson[1], Carol Tishelman[2,3], Kerrie Noonan [4,5], Rosemary Leonard[5], Lars E. Eriksson[1,6,7], Ida Goliath[1,8¶], Joachim Cohen[9¶]

[1] Department of Neurobiology, Care Sciences and Society, Karolinska Institutet, Huddinge, Sweden

[2] Department of Learning, Informatics, Management and Ethics, Karolinska Institutet, Solna, Sweden

[3] Stockholm Health Care Services, Region Stockholm, Sweden

[4] Translational Health Research Institute, Western Sydney University, Sydney, Australia

[5] Death Literacy Institute, Australia

[6] School of Health Sciences, City, University of London, United Kingdom

[7] Medical Unit Infectious Diseases, Karolinska University Hospital, Huddinge, Sweden

[8] Stockholm Gerontology Research Center, Stockholm, Sweden

[9] End-of-Life Care Research Group, Vrije Universiteit Brussel and Ghent University, Brussels, Belgium

*Corresponding author: Therese Johansson

E-mail: therese.johansson@ki.se (TJ)

¶ These authors contributed equally to this work.

Abstract

The death literacy index (DLI) was developed in Australia to measure death literacy, a set of experience-based knowledge needed to understand and act on end-of-life (EOL) care options but has not yet been validated outside its original context. The aim of this study was to develop a culturally adapted Swedish-language version of the DLI, the DLI-S, and assess sources of evidence for its validity in a Swedish context.

The study involved a multi-step process of translation and cultural adaptation and two validation phases: examining first content and response process validity through expert review (*n*=10) and cognitive interviews (*n*=10); and second, internal structure validity of DLI-S data collected from an online cross-sectional survey (*n*=503). The psychometric evaluation involved analysis of descriptive statistics on item and scale-level, internal consistency and test-retest reliability, and confirmatory factor analysis.

During translation and adaptation, changes were made to adjust items to the Swedish context. Additional adjustments were made following findings from the expert review and cognitive interviews. The content validity index exceeded recommended thresholds. The psychometric evaluation provided support for DLI-S' validity. The hypothesized six-factor model showed good fit. High internal consistency reliability was demonstrated for the overall scale (Cronbach's α=0.94) and each sub-scale (α 0.81–0.93). Test-retest reliability was acceptable, ICC ranging between 0.66–0.85.

Through a comprehensive assessment of several sources of evidence, we show that the DLI-S demonstrates satisfactory validity and acceptability to measure death literacy in the Swedish context. There are, however, indications that the sub-scales measuring community capacity perform worse in comparison to other scales and may function differently in Sweden than in the original context. The DLI-S has potential to contribute to research on community-based EOL interventions.

Keywords

Instrument development; Public Health; Questionnaire Surveys; Reliability and Validity; Death education; End-of-Life Care

Introduction and aim

Just as health literacy relates to the extent to which people can find, interpret, and use health information and services [1], death literacy (DL) is a newly-coined term denoting knowledge and skills needed to understand and act on options for end-of-life (EOL) and death care [2]. Building on new public health approaches that highlight individual and community capacity-building and empowerment to handle issues of dying, death, and loss [3], DL is theorized to develop from engaging with EOL care and learning from those experiences [4].

The *death literacy index* (DLI), an instrumentalization of DL, is a questionnaire developed from research in Australia about people's experiences of EOL care [5]. DLI is intended to measure population levels of DL and evaluate EOL-related educational initiatives [2]. As such, the DLI may constitute a promising tool for appraising impact of health promoting activities in relation to EOL issues. However, as the DLI has only been used to date among adults in the Australian general public, the extent to which DL and its operationalization are comparable across cultures is unknown. While the DLI has demonstrated high convergent validity and reliability in Australia [2, 6], it has not yet been translated into or validated in other languages or outside the Australian context. The aim of this study was to develop and culturally adapt Swedish-language version of the DLI (DLI-S) and assess its validity in a Swedish context.

Materials and methods

Study design

As illustrated in Figure 1, this instrument validation study used a multi-step mixed methods approach comprising three phases, to translate and culturally adapt the Death Literacy Index (DLI) into Swedish and to assess the validity of the resulting DLI-S.

Fig 1. Schematic of the study process.

Steps in the translation and adaptation of the DLI-S
Sources of validity evidence

The Death Literacy Index

The DLI is a multi-dimensional instrument containing 29 items distributed over four dimensions of DL; *Practical knowing* (n of items=8), *Learning from Experience* (n=5), *Factual knowledge* (n=7), and *Community capacity* (n=9) [2]. The dimensions are represented in the DLI as four scales, two of which contain sub-scales capturing specific dimensional facets; *Practical knowing* has the sub-scales *Talking support* (n=4) and *Hands-on care* (n=4), whereas *Community Capacity* has the sub-scales *Accessing help* (n=5) and *Community support groups* (n=4). DLI items are in the form of statements with ordered category responses on a 5-point Likert-type scale, usually ranging from "do not agree at all" and "strongly agree". Since the DLI covers various aspects of DL, scores are calculated for each scale and sub-scale, using transformed mean scores. Total DL is calculated as a higher-order composite score, which is said to reflect overall capacity gained from previous experiences [6]. The original DLI and items in the DLI-S are presented in S1 and S2 Files respectively.

Translation and adaptation of the Swedish Death Literacy Index

After obtaining permission to translate the DLI from the original developers, co-authors RL and KN, the DLI-S was developed following Beaton et al.'s [7] recommendations for instrument translation and cross-cultural adaptation. The 29 items with corresponding instructions and response categories were independently translated from English to Swedish by co-authors TJ and ÅO, both native Swedish speakers proficient in English. The two initial forward translations were compared to identify discrepancies and discuss ambiguous wordings, resulting in a joint draft [7]. This DLI-S draft was sent to the members of a multidisciplinary research group with both native English and Swedish speakers, who individually reviewed the draft, prior to continued revision through a process of negotiated consensus in a series of meetings [8] after which changes to content were decided so that the

Swedish items would convey the same conceptual understanding as the original instrument [7, 9]. Back translation was not used in this study as it has been criticized for missing variation in linguistic meaning and cultural nuances [10].

Instrument validation

Validity is defined as a unitary concept assessed from several sources of evidence, each contributing to the overall validity of an instrument [11]. Validation involves the assessment of evidence to support interpretations of scores in relation to the intended use of an instrument [12]. Validity is context-specific, and thus, validation is strongly recommended whenever an instrument is to be used in a new, qualitatively different, population or context [13]. In this article we focus on three sources of validity evidence: 1) *Evidence based on content*, relating to the adequacy and relevance of items to represent and score the construct measured [14]; 2) *Evidence based on response processes*, which involves exploration of respondents' actions and cognitive processes to identify possible sources of error, e.g., challenges with interpreting and answering items [11]; and 3) *Evidence based on internal structure*, which concerns how items relate to each other and to the overarching construct, often measured as reliability across items, time, or respondents [14].

Phase 1. Validity evidence based on content and response process

1.1 Expert panel review

Procedure and participants

An expert review was conducted to evaluate the validity of DLI-S items to measure DL [15]. Since DLI targets the general population, there is no delimited area of expertise relevant for this step. We therefore made efforts to recruit ten panel members with varying ages, backgrounds, and personal and professional perspectives in relation to the EOL, e.g., palliative care, gerontology, ethnology, professional translation, clinical nursing, and from patient interest organizations. Email invitations were sent with information about the study purpose and methods. Inclusion criteria were proficiency in both Swedish and English. Each panel member reviewed the DLI-S independently, using an online

survey, accessible only after providing informed consent. Following recommendations by Grant and Davis (15), panel members were first provided with a summary description of the conceptual DL model and provided with information about the intended use of the DLI. As shown in S3 File, the review comprised two main sections: *Translation review***,** in which the semantic and cultural equivalence between each Swedish item and the original corresponding English-language item was assessed on a four-point scale; and *Content validity assessment*, in which the DLI-S items' relevance and clarity of content were rated on a four-point scale. Panel members could comment and suggest changes for every item throughout both sections of the survey.

Data analysis

Comments related to the translation and content of each item were reviewed and summarized by TJ. Quantitative ratings were collated in a matrix in Microsoft Excel to calculate the content validity index (CVI), i.e., inter-rater agreement at the item-level (I-CVI) and scale-level (S-CVI). I-CVI represents the proportion of panel members rating an item positively (e.g., 3 or 4) and is recommended to be at least 0.78 [16]. S-CVI was calculated using average proportion, recommended for panels of ≥8 [17].

1.2 Pre-testing using cognitive interviews

Procedure and participants

To determine whether the DLI-S items were acceptable, comprehensible, and able to generate information as intended, authors TJ and ÅO conducted cognitive interviews with a new sample from the target population [18], i.e., adults from the general public. Interviews combined think-aloud technique, in which the respondent describes their reasoning out loud as they read and respond to each item [19], and verbal probing, whereby the interviewer asks questions to clarify and further explore any issues [20]. Participants were recruited through convenience sampling in the researchers' networks, striving for variation in terms of age, gender, educational level, and professional background. Due to the Covid-19 pandemic, interviews were conducted online using

Zoom. All participants received written information about the study in advance and consented orally to participate in audio-recorded interviews. While we assessed the risk of harm to participants as low, the interviewers were experienced in handling emotional reactions and could refer participants to other sources for support if needed.

Data analysis

Data were based on the interviewers' field notes, using audio-recordings as back-up if needed, and compiled into a summary matrix, linking comments to the items and sub-scales to which they referred [21]. The matrix was reviewed in recurring consensus meetings to inform decisions regarding item retention, revision, or deletion and modifications to instructions or response categories.

1.3 Literacy review

To investigate linguistic accessibility, the lexical profile of the DLI-S was reviewed by an independent consultant using the software *AntWordProfiler* to examine the proportion of words among the 5,000 most common in Swedish. This was done on two occasions: after the first 5 cognitive interviews and again after the DLI-S had been finalized (Figure 1).

Phase 2. Validity evidence based on internal structure

Procedure and participants

Data was collected from September–November 2021 through an online survey administered by an external data collection agency with a pre-existing panel of ca 100,000 Swedish adults willing to partake in surveys on various topics. Since this study aimed for theoretical generalization rather than making statistical inferences regarding population estimates of death literacy, a representative probability sample was not necessary [22]. Still, we strove for a sample reflective of the heterogeneity within the Swedish population. Survey invitations were sent to a quota sample (*n*=2991), stratified by gender, age, and region, from the agency's existing panel. The minimum sample size was set to 500 to have sufficient data and power for confirmatory factor analysis (CFA)

[23]. The survey comprised the DLI-S items and questions about sociodemographic variables and EOL experiences and was only accessible to panel members who agreed to participate after being informed about the study. Participants were notified that they could exit the survey at any time and were provided with the researchers' contact information in case they needed support or had questions or comments following participation. At the end of the survey, participants were asked whether they were willing to complete the survey a second time, to assess test-retest reliability. Of the 412 that agreed to participate in a follow-up, 82 were re-invited to the survey. The retest sample size was set to minimum 50 to provide sufficient for calculating intra-class correlation coefficients (ICC) [24]. The time interval was set to approximately 4 weeks, chosen to allow enough time to avoid rehearsal effects but short enough to minimize the risk of participants experiencing real change that might alter their responses [25].

Data analysis

Statistical analyses were conducted using IBM SPSS Statistics 28 and the *lavaan* package [26] in R (version 4.1.1). Descriptive statistics were used to explore socio-demographic characteristics of the sample and analyze central tendencies and dispersion on item- and scale-level. Response variation was examined at item-level by investigating if all five response categories were used and at scale-level by identifying whether there were floor or ceiling effects, i.e., ≥15% of responses in the maximum and/or minimum category [27]. Inter-item correlations were calculated for all items.

Corrected item-total correlations were examined and values between 0.2–0.7 were considered good discrimination within a scale [28]. Internal consistency reliability was assessed by calculating Cronbach's α coefficients and confidence intervals of items, sub-scales and the full DLI. Values ≥0.8 were considered as demonstrating good reliability [29]. However, since the appropriateness of Cronbach's α as a sole measure of reliability has been questioned, due to the effects of the number of scale items and assumptions of unidimensionality and tau equivalence [30, 31], we also calculated average inter-item correlation (AIC), a more robust indicator of internal consistency [32]. The acceptable range for AIC is considered to be 0.15–0.5 [29]. ICC was calculated to assess scale test-

retest reliability, using the two-way mixed effects, single measure model (ICC type 3,1). ICC >0.9 indicates excellent reliability, whereas ICC >0.75 is considered good, >0.5 is moderate and <0.5 suggests poor reliability [33].

CFA was used to assess the fitness of the DLI factor structure. Three factor models were tested: with DLI treated as one universal factor; as four factors (corresponding to the original main DL dimensions); and as six factors (corresponding to four sub-scales (*1.1, 1.2, 4.1, 4.2*) and two dimension scales (*2* and *3*). As the 5-point response range used in the DLI was relatively short, data were modelled as ordinal rather than continuous. Diagonally weighted least squares (DWLS) were used as model estimator, since this has been shown to perform better than robust maximum likelihood estimation with ordinal data [34]. Tucker-Lewis index (TLI) and Comparative Fit Index (CFI) >0.95, root mean square error of approximation (RMSEA) >0.06, Standardized Root Mean Square Residual (SRMR) >.08, and χ^2 *p* value >.05 were used as indices of good model fit [35].

Ethical considerations of the study

The study process described below was approved by the Swedish Ethics Review Authority (reference number 2021-00915) and conducted according to the ethical principles of the Helsinki Declaration [36]. All study participants received written information about the nature of the study, including its subject, purpose, and procedure, as well as their right to withdraw. Informed consent to participate was obtained from all participants. The DLI asks about experiences related to the EOL, a potentially sensitive topic. However, previous research has shown that questions addressing dying or death may have an effect on immediate mood but unlikely to cause harm [37]. Furthermore, it should be noted that participants were not persons known to be at the EOL themselves.

Results

Translation and adaptation of the DLI-S

Minor changes were made to item content to ensure conceptual equivalence to the original items and better adapt items to the Swedish context. Detailed examples of changes made at various stages of the item revision process during both translation/adaptation and validation phases are provided in S4 Table, using two DLI-S items as examples.

Phase 1. Validity based on content and response process

1.1 Expert review

All ten individuals contacted agreed to participate, of whom seven were women. The qualitative data, e.g., potential concerns identified and suggestions for improvement, were addressed in discussions about item revision during consensus meetings with the research group, thereby informing the continued process of revising the DLI-S. Overall, the expert review identified words and terms that were awkward or unclear in Swedish and raised questions regarding content relevance in Sweden. To address these issues, minor changes to content were made for several items (see S4 Table). I-CVI scores ranged between 0.837–0.987, indicating that each item was considered relevant for the DL construct. The full DLI-S demonstrated good content validity with an $S\text{-}CVI_{Ave}$=0.926. All CVI scores are presented in supplement table S5.

1.2 Cognitive interviews

In total, ten people (seven women) participated in the cognitive interviews. Overall, the cognitive interviews showed that some items could raise memories of past experiences. Nevertheless, DLI-S content was generally acceptable to participants, i.e., not distressing, interesting, and with items of varying difficulty. Table 1 presents six types of issues requiring minor changes to the DLI-S and affected items and/or scales, based on participants' response processes, comments about the instrument, and suggestions for improvement (see S4 Table for more detailed examples of item

revision). For example, we found that participants' responses to the question in sub-scale *Talking Support* (1.1), "*how difficult or easy you would find the following* [items]", indicated that the question did not sufficiently prompt participants to think about their self-perceived competence or preparedness for engaging in conversations about EOL issues when answering, as was intended. Instead, they often mentioned a combination of values, perceived social taboos, or relation to the conversation partner when thinking aloud about their responses to the items. Consequently, the question was reformulated to "*how prepared would you be to talk about the following* [conversations about EOL issues]*?*", to better guide respondents to consider their readiness to engage in conversations about the EOL when answering items in this sub-scale.

Table 1. Overview of issues identified in the cognitive interviews and how they were addressed.

Issue	Affected items/ scales	Revisions to address issue
Vague or ambiguous item statement	4, 13, 14, 16,19, 24	Items clarified to better reflect conceptual meaning and/or semantic precision
Double-barrelled item, e.g., item statement contains more than one behaviour/trait	19	First section removed to make item statement more concise and focused on the trait in question
Item relevance or applicability to Swedish context	14, 15, 20, Scale 4.1	Examples provided clarify content (21); item wordings revised and refined (14, 15, 21). Question in scale 4.1 rephrased to better suit the Swedish context with universal health care
Overlapping items (item content is perceived as repeating)	Scale 2	No change at this stage, all items retained as they are considered to contribute with different aspects on a theoretical level
Unclear question format	Sub-scales 1.1 and 4.1	Questions specified to better reflect intended use of the instrument; clarifying instructions added for the instrument to highlight that questions relate to individual perceptions and that there are no right or wrong answers

Unsuitable or unclear response categories	Sub-scales 1.1 and 1.2	Response categories changed to better suit the scale question (from *very difficult/very easy* to *not prepared at all/very prepared*)

1.3 Literacy review

The first review found that 84.7% of the words used in the DLI-S were among the 5,000 most common in Swedish. The consultant suggested more common or easy-to-read alternatives to difficult or uncommon terms, which formed the basis for another round of item revision through consensus meetings in the research group. This revised DLI-S version was then used in the five subsequent cognitive interviews. A second literacy review was conducted on the final DLI-S, in which the rate had increased to 93.3%, which was deemed sufficient.

Phase 2. Validity based on internal structure

In total, 503 people completed the survey, giving a response rate of 17%. At the retest, 55 participants completed the survey, giving a second response rate of 67%. Socio-demographic characteristics of participants are presented in Table 2. Since the online survey used a mandatory response procedure, requiring all items to be answered to proceed, there were no missing values in the data. Item-level descriptives are presented in Table 3. Inter-item correlations within sub-scales were generally high, e.g. >0.5, and presented in supplement table S6.

Table 2. Sample characteristics of participants in Phase 2 (*n*=503)

Socio-demographic characteristics	Mean (SD)	Range
Age	49.95 (17.92)	18-86
	**Count**	**Percentage**
**Gender**		
Male	253	50.4%
Female	246	49.0%
Other (Non-binary or trans)	3	0.6%
**Highest level of completed education**		
Lower secondary education or less	42	8.3%
Upper secondary education	207	41.2%
Post-secondary education	24	4.8%
Higher general or vocational education diploma	77	15.3%
Higher education, bachelor's degree or equivalent	89	17.7%
Higher education, master's degree or more	64	12.7%
**Origin**		
Sweden	467	92.8%
Europe, excl. Sweden	26	5.2%
Outside Europe	10	2.0%
**Work or volunteering experience**		
EOL care provision (work / volunteer)	71 / 28	14% / 5.6%
Grief support (work / volunteer)	49 / 32	9.9% / 6.2%
**Professional experience in care**		
Care sector	60	11.9%
Social care sector	39	7.8%
Both care and social care	12	2.4%
No professional experience	392	77.9%
**EOL experiences** [a]		
Death of a family member, close relative, or friend	408	81.2%
Own life-threatening illness	45	9.0%
Supporting a person with a life-threatening illness	113	22.4%
Care for a relative at the EOL	64	12.8%
Providing EOL care professionally	55	10.9%
No EOL experience	29	5.8%

Notes: EOL=end-of-life; [a] Multiple responses allowed, sum≠503

Table 3. Descriptive statistics for the DLI-S items* (*n*=503).

Items	Mean	SD	Corrected item-total correlation
1. Talk about dying, death, or grief with a close friend	3.91	1.04	.43
2. Talk about dying, death, or grief with a child	2.98	1.27	.40
3. Talk with a bereaved person about their loss	3.58	1.11	.53
4. Talk with care staff about support for a person who will die at home or in their place of care	3.66	1.12	.61
5. Feed or help someone to eat	3.75	1.21	.51
6. Wash someone	3.09	1.39	.55
7. Lift someone or help to move them	3.64	1.27	.38
8. Administer injections	2.55	1.51	.46
9. Made me more emotionally prepared to support others with processes related to death and dying	3.56	1.06	.57
10. Made me think about what is important and not important in life	3.98	0.99	.43
11. Made me wiser and given me new understanding	3.70	0.96	.49
12. Increased my compassion toward myself	3.38	1.05	.38
13. Made me better prepared to face similar challenges in the future	3.60	1.02	.58
14. I know about rules and regulations regarding deaths at home	2.12	1.18	.66
15. I know that there are documents that can help a person plan before death	3.28	1.38	.47
16. I know enough about how [the health and social care systems] operate to be able to support a person in receiving care at the end of life	2.60	1.29	.75
17. I know about processes for funerals, where I can turn, and which choices are available	3.26	1.31	.58
18. I know how to access palliative care in the area where I live	2.27	1.33	.69
19. I know enough to make decisions about medical treatments and understand how they may affect quality of life, at the end of life	2.53	1.33	.70
20. I am aware of different ways that cemetery staff can be of help around funerals	2.67	1.25	.64
21. To get support in the area where I live, e.g., from clubs, associations, or volunteer organizations	2.12	1.16	.68
22. To get help with providing day to day care for a person at the end of life	2.48	1.31	.74
23. To get equipment that are required for care	2.69	1.36	.69
24. To get support that is culturally appropriate for a person	2.06	1.15	.63
25. To get emotional support for myself	2.46	1.25	.72
26. People with diseases that might lead to death	2.83	1.28	.66
27. People who are nearing the end of their lives	2.61	1.28	.70
28. People who are caring for someone who is dying	2.50	1.29	.72
29. People who are grieving	2.86	1.32	.64

* DLI-S items translated to English

Reliability

The DLI-S demonstrated high internal consistency reliability, Cronbach's α=0.94 for the overall scale and between 0.81–0.93 for the sub-scales. Test-retest reliability was moderate to good, with scale-level ICC ranging 0.66–0.84. Reliability estimates for the full DLI-S and each sub-scale are presented in Table 4.

Confirmatory factor analysis

Fit indicators for all tested models are presented in Table 5. The one-factor model was tested first, demonstrating adequate fit. The four-factor model, corresponding to the main dimensions of DL, generated a better fit, though still not meeting all recommended thresholds. The six-factor model showed good fit, performing best of the three models tested. As illustrated in Figure 2, factor loadings in this model were generally high, ranging between 0.57–0.95, and correlations between factors were moderate to strong, between 0.43–0.86.

Table 4. Descriptive statistics and reliability estimates for the full DLI-S and each sub-scale.

	Mean score (SD)[a]	Floor/ceiling effect (%)	Cronbach's α (95% CI)[b]	AIC[b]	ICC (95% CI)[c]
DLI-S (full scale)	5.15 (1.86)	0.2/0.6	0.94 (0.94-0.95)	0.36	0.85 (0.76-0.91)
Talking support	6.28 (2.28)	0.8/8.9	0.82 (0.78-0.84)	0.52	0.68 (0.50-0.80)
Hands-on care	5.63 (2.69)	1.6/10.3	0.81 (0.78-0.84)	0.53	0.81 (0.69-0.88)
Learning from experience	6.59 (2.05)	0.2/8.7	0.83 (0.84-0.88)	0.56	0.66 (0.49-0.79)
Factual knowledge	4.13 (2.49)	3.2/2.0	0.89 (0.87-0.90)	0.53	0.84 (0.73-0.90)
Accessing help	3.34 (2.71)	18.5/2.0	0.92 (0.91-0.93)	0.69	0.72 (0.57-0.83)
Community support groups	4.21 (2.89)	13.1/6.2	0.92 (0.91-0.93)	0.74	0.67 (0.50-0.79)

Notes: [a]Mean scores are transformed to a range from 0-10; [b] *n*=503; AIC=Average inter-item correlation; ICC= Intra-class correlation; [c] *n*=55

Table 5. Fit indicators of tested factor models of death literacy (*n*=503).

**Tested model** (Estimator DWLS)	CFI	TLI	RMSEA (CI)	SRMR	χ^2/df
One factor	0.933	0.928	0.200 (0.196-0.204)	0.139	7966.767/377***
Four factors	0.980	0.978	0.110 (0.106-0.114)	0.081	2629.432/371***
Six factors	0.993	0.993	0.064 (0.060-0.068)	0.054	1107.631/362***

Notes: DLWS=Diagonal weighted least squares; TLI=Tucker-Lewis Index, CFI=Comparative Fit Index
***p <0.001

Fig 2. Path diagram of the best-fitting model, demonstrating standardized factor loadings for items and correlations between factors.

Factor loadings

Correlation coefficients between factors

Scale descripti

Scale descriptives, i.e., mean scores, standard deviations, and floor and/or ceiling effects, are presented in Table 4. Total DLI scores and sub-scale scores were normally distributed in the sample, with the exception of the sub-scale *Accessing help*, which demonstrated a floor effect, i.e., a negatively skewed distribution.

Discussion

This mixed-methods study is, to our knowledge, the first that has assessed the validity of the DLI in a new language, outside its original Australian context. Using an iterative multi-step process of translation, adaptation, and validation that generated both qualitative and quantitative data, we performed a comprehensive assessment of several sources of validity evidence for the DLI-S in a new context with regard to culture, language, and care organization and provision. The results found evidence of cross-cultural validity of the DLI and support for the proposed six-factor model of DL.

Both I-CVI and S-CVI ratings exceed the recommended minimum set out by Polit, Beck (16), suggesting support for DLI-S' validity in terms of item clarity and relevance for the DL construct. Despite high CVI ratings, several potential issues were raised by experts regarding item meaning and suitability in a Swedish context, highlighting the need for qualitative data to make meaningful assessments of content validity for translated instruments. Likewise, qualitative findings from the cognitive interviews were instrumental for guiding the researchers in addressing problematic items and unclear instructions. The cognitive interviews showed that the DLI-S could be completed by people with varying EOL experiences, from those with who describe themselves as having very limited EOL experiences to experts in the field. Importantly, the cognitive interviews also

demonstrated that the DLI-S was not perceived as too sensitive or distressing, although it was noted that certain questions could bring up emotional memories. Several participants mentioned that they thought the items were interesting and thought-provoking, suggesting that completing the DLI-S could constitute a positive self-reflective experience. This is important since death education often emphasizes the role of reflection and sharing of experiences as part of the learning process [38]. In addition, the high proportion of survey participants who were willing to complete the survey a second time further illustrate DLI-S' acceptability. This is a significant finding since death is often described as a taboo topic in Sweden [39]. Still, as Arthur, Menon (40) state, cognitive interviews about instruments of a sensitive nature can be challenging: it may be difficult to raise concerns about intrusive or insensitive questions in a face-to-face situation, where a participant might feel obliged to justify their opinion.

One challenge regarding content validity concerns the definition of "community" used in item 21 (*Accessing help)*. There is no Swedish word for "community", which could be translated with an emphasis on either social, geographical, or cultural connotations. To guide the translation process, the Swedish research team discussed the intention of the term with the original DLI developers. Based on this discussion, we used a translation that highlight location, i.e., neighborhood. Even if this is a common interpretation of the term and no major problems were identified during the cognitive interviews, it is possible that this translation was too narrow and influenced how the question functioned in the Swedish setting, as it is more specific than the English term.

The DLI-S was found to have satisfactory psychometric properties, with support for validity evidence based on internal structure. However, the findings also identify some potential issues with the DLI-S that are important to consider. High Cronbach's α for all scales and sub-scales indicate that items are inter-related but does not necessarily mean that the scale is unidimensional [29]. Scale-level AICs further confirm strong item inter-relatedness, with values exceeding the recommended range. This finding raises questions of item redundancy, i.e., presence of items that do not sufficiently contribute

with new information to measure the construct. The inter-item correlations suggest that the DLI-S might benefit from having one or several items removed: in particular the sub-scales comprising the dimensional scale *Community capacity* (*Accessing help* and *Community support groups*). These sub-scales consistently performed worse in comparison to the other scales and the instrument overall. For example, the floor effect in *Accessing help* indicates that the sub-scale has limited ability differentiating between responses at low levels, which might reduce reliability [41]. This finding also points to differences between the Swedish and Australian context that appear as variation in item difficulty for these items [42]. In addition, the confirmatory factor analysis showed that two factors (*Existing knowledge* and *Accessing help*) were highly correlated, suggesting that items in these scales may measure one, underlying, factor rather than two distinct dimensions of DL. Further studies are thus warranted to explore the performance of a shorter DLI-S version in the Swedish context and to investigate if a five-factor model constitutes a better fit for the DLI-S and the extent to which this might be relevant in other contexts.

In sum, the validity evidence for internal structure show that the DLI-S performs well psychometrically, although the comparatively worse performance of the *Community capacity* sub-scales may indicate difference in function or meaning in Sweden compared to Australia. This finding could be an accurate reflection of differences between the Swedish and Australian context, particularly in how care systems are organized and people's expectations of and interactions with them. In Sweden, public awareness of palliative and EOL care has been found to be generally low [39]. Unlike many other countries, Swedish EOL care is not dependent on public involvement such as volunteerism and charitable donations [43, 44]. Instead, Sweden has a long history of tax-funded universal welfare and high levels of trust in health care providers and institutions [45], which has remained stable even during the Covid-19 pandemic [46]. Indeed, participants in the cognitive interviews who gave low ratings for items in these sub-scales described feeling confident in their belief that if needed, they could turn to their primary care clinic for support or contact the national

hub for information about health and healthcare services in Sweden that is accessible round-the-clock by phone or chat.

Methodological discussion

There are several methodological limitations that should be acknowledged. Participants in the online survey comprise a non-probability quota sample that was recruited from an existing national panel. Although the composition is balanced to that of the Swedish population in terms of age, gender, and place of residence; the sample is slightly positively skewed compared to the population average [47] and underrepresented concerning place of birth, as 19.7% of the Swedish population are born outside Sweden, compared to only 7.2% of our participants [48]. More importantly, non-probability sampling raises concerns of possible self-selection and disproportion in unmeasured characteristics that may produce biased results, particularly if the purpose is making population estimates and representativeness [49, 50]. However, as theoretical rather than statistical generalization was the aim of this validation study, a representative and random sample was not required. Similarly, our 17% response rate may be considered low, but a high response rate is not necessary for the purpose of validating an instrument. Still, additional studies using larger and representative samples may allow further examination of the generalizability of the DLI-S' validity in the Swedish population. In addition, it was not possible to examine convergent validity in relation to other instruments in this study since Swedish translations of other validated instruments measuring comparable constructs are lacking.

Despite these limitations, the study complied with gold standard practice for instrument translation, cultural adaptation, and validation, applying a rigorous process to assess validity and reliability evidence. It should be highlighted that since the aim of the study was to assess the validity of the DLI-S and not to develop a modified DLI version, no items were removed even if there were some indications of items that could be challenging in terms of comprehensibility (in phase 1) or may be redundant for measuring a DL dimension (in phase 2). Using bilingual field researchers instead of a

professional translator during forward translation can be considered a strength, as proposed by Nolte et al. [51] who point out that professional translators often focus on the accuracy of the linguistic translation rather than general readability and conceptual meaning. We also made efforts to address previously identified issues of transparency in validation, e.g., providing full instructions for the expert panel review (S3 File) to increase clarity regarding the basis of ratings [15]. An additional strength in this study is the use of literacy reviews, which is imperative for identifying possible unfair and unintended advantages or disadvantages to certain groups in the target population that might otherwise affect an instrument's usefulness [52].

Implications

Rather than a measure of knowledge and skills alone, DL seems to represent a more overarching familiarity with the dying process, as recently suggested by Hayes et al. [53], that also encompasses attitudes and self-efficacy. This perspective seems fitting in the Swedish context, as our findings suggest that alongside gauging the extent of knowledge gained from prior EOL experiences, the DLI appears to capture perceived capacity to handle EOL-related issues and confidence in abilities to learn. The demonstrated acceptability and good psychometric properties of the DLI-S suggest that it has potential to be used as an instrument to measure DL on national and local levels in Sweden. Nevertheless, more research is needed to better understand the DL construct, particularly across cultures. Furthermore, the suitability of the DLI as an evaluation tool for EOL-related educational initiatives, both within and outside formal care settings, needs to be examined in Sweden and elsewhere.

Lack of public awareness of EOL care and civic preparedness for engaging with issues related to death and dying has been identified as hindering people's access to high-quality care [54]. Internationally, there is a growth of community based EOL interventions, such as compassionate communities, which are intended to encourage people to assist and support those at the EOL within their community. With further validation, the DLI has potential to be a multifaceted instrument appropriate for

continued cross-cultural research and better understanding of impact of such initiatives [55]. This is increasingly pertinent as it is expected that EOL care provision will progressively take place outside formal care settings, e.g., aging populations, both internationally [56, 57] and in Sweden [58]. Additional research can also shed light on whether the DLI may be useful in the care context to measure overarching competence for EOL care among staff, especially to evaluate more integrated and comprehensive EOL education interventions [59, 60].

Conclusion

This study provides empirical evidence supporting the validity of the Swedish translation and adaptation of the 29-item DLI to measure death literacy in the adult general public. The DLI-S was shown to be acceptable and feasible to answer regardless of the extent of respondents' prior EOL experiences. In a time with growing interest in building community preparedness for EOL issues, the DLI-S constitutes a promising instrument with good properties to measure overall capacity to engage with EOL care. Even though the six-factor model of the DLI yielded a good fit, our results show some characteristics that could potentially impact its measurement properties in a Swedish context.

Acknowledgements

The authors would like to acknowledge the generous contributions by Aleksandra Sjöström-Bujacz, Leo Kowalski, and Sara Runesdotter to the statistical analyses. We also acknowledge the contribution of Terese Stenfors during the translation of the DLI-S.

References

1. Kindig DA, Panzer AM, Nielsen-Bohlman L. Health literacy: a prescription to end confusion. In: Medicine Io, editor. Washington DC: The National Academies Press; 2004.

2. Leonard R, Noonan K, Horsfall D, Kelly M, Rosenberg JP, Grindrod A, et al. Developing a death literacy index. Death Stud. 2021:1-13. doi: 10.1080/07481187.2021.1894268.

3. Sallnow L, Tishelman C, Lindqvist O, Richardson H, Cohen J. Research in public health and end-of-life care – Building on the past and developing the new. Prog Palliat Care. 2016;24(1):25-30. doi: 10.1080/09699260.2015.1101260.

4. Noonan K, Horsfall D, Leonard R, Rosenberg J. Developing death literacy. Prog Palliat Care. 2016;24(1):31-5. doi: 10.1080/09699260.2015.1103498.

5. Horsfall D, Noonan K, Leonard R. Bringing our dying home: How caring for someone at end of life builds social capital and develops compassionate communities. Health Sociology Review. 2012;21(4):373-82. doi: 10.5172/hesr.2012.21.4.373.

6. Leonard R, Noonan K, Horsfall D, Psychogios H, Kelly M, Rosenberg J, et al. Death Literacy Index: A Report on its Development and Implementation. Sydney: Western Sydney University: 2020.

7. Beaton DE, Bombardier C, Guillemin F, Ferraz MB. Guidelines for the process of cross-cultural adaptation of self-report measures. Spine. 2000;25(24):3186-91.

8. McGreevy J, Orrevall Y, Belqaid K, Bernhardson BM. Reflections on the process of translation and cultural adaptation of an instrument to investigate taste and smell changes in adults with cancer. Scand J Caring Sci. 2014;28(1):204-11. Epub 2013/02/07. doi: 10.1111/scs.12026. PubMed PMID: 23383751.

9. Beck CT, Bernal H, Froman RD. Methods to document semantic equivalence of a translated scale. Research in Nursing & Health. 2003;26(1):64-73.

10. Geisinger KF. Cross-cultural normative assessment: translation and adaptation issues influencing the normative interpretation of assessment instruments. Psychol Assess. 1994;6(4):304-12.

11. American Educational Research Association, American Psychological Association, National Council on Measurement in Education. Standards for educational and psychological testing. Washington DC: American Educational Research Association; 2014.

12. Zumbo BD, Chan EK. Setting the stage for validity and validation in social, behavioral, and health sciences: Trends in validation practices. Validity and validation in social, behavioral, and health sciences: Springer; 2014.

13. Gjersing L, Caplehorn JR, Clausen T. Cross-cultural adaptation of research instruments: language, setting, time and statistical considerations. BMC Med Res Methodol. 2010;10(1):13. doi: 10.1186/1471-2288-10-13.

14. Cook DA, Zendejas B, Hamstra SJ, Hatala R, Brydges R. What counts as validity evidence? Examples and prevalence in a systematic review of simulation-based assessment. Advances in Health Sciences Education. 2014;19(2):233-50.

15. Grant JS, Davis LL. Selection and use of content experts for instrument development. Research in nursing & health. 1997;20(3):269-74.

16. Polit DF, Beck CT, Owen SV. Is the CVI an acceptable indicator of content validity? Appraisal and recommendations. Res Nurs Health. 2007;30(4):459-67. Epub 2007/07/27. doi: 10.1002/nur.20199. PubMed PMID: 17654487.

17. Polit DF, Beck CT. The content validity index: are you sure you know what's being reported? Critique and recommendations. Research in nursing & health. 2006;29(5):489-97.

18. Beatty PC, Willis GB. Research Synthesis: The Practice of Cognitive Interviewing. Public Opin Q. 2007;71(2):287-311. doi: 10.1093/poq/nfm006.

19. Fonteyn ME, Kuipers B, Grobe SJ. A Description of Think Aloud Method and Protocol Analysis. Qual Health Res. 1993;3(4):430-41. doi: 10.1177/104973239300300403.

20. Willis GB, Miller K. Cross-Cultural Cognitive Interviewing. Field Methods. 2011;23(4):331-41. doi: 10.1177/1525822x11416092.

21. Willis GB. Analysis of the cognitive interview in questionnaire design: Oxford University Press; 2015.

22. Pasek J. When will Nonprobability Surveys Mirror Probability Surveys? Considering Types of Inference and Weighting Strategies as Criteria for Correspondence. International Journal of Public Opinion Research. 2016;28(2):269-91. doi: 10.1093/ijpor/edv016.

23. Mundfrom DJ, Shaw DG, Ke TL. Minimum Sample Size Recommendations for Conducting Factor Analyses. International Journal of Testing. 2005;5(2):159-68. doi: 10.1207/s15327574ijt0502_4.

24. Shoukri MM, Asyali M, Donner A. Sample size requirements for the design of reliability study: review and new results. Statistical Methods in Medical Research. 2004;13(4):251-71.

25. Polit DF. Getting serious about test-retest reliability: a critique of retest research and some recommendations. Qual Life Res. 2014;23(6):1713-20. Epub 2014/02/08. doi: 10.1007/s11136-014-0632-9. PubMed PMID: 24504622.

26. Rosseel Y. lavaan: An R Package for Structural Equation Modeling. Journal of Statistical Software. 2012;48(2):1-36.

27. McHorney CA, Tarlov AR. Individual-patient monitoring in clinical practice: are available health status surveys adequate? Qual Life Res. 1995;4(4):293-307. doi: 10.1007/bf01593882.

28. Streiner DL, Norman GR, Cairney J. Health measurement scales: a practical guide to their development and use: Oxford University Press, USA; 2015.

29. Clark LA, Watson D. Constructing validity: New developments in creating objective measuring instruments. Psychol Assess. 2019;31(12):1412-27. Epub 2019/03/22. doi: 10.1037/pas0000626. PubMed PMID: 30896212; PubMed Central PMCID: PMCPMC6754793.

30. Sijtsma K. On the use, the misuse, and the very limited usefulness of Cronbach's alpha. Psychometrika. 2009;74(1):107.

31. Dima AL. Scale validation in applied health research: tutorial for a 6-step R-based psychometrics protocol. Health Psychology and Behavioral Medicine. 2018;6(1):136-61. doi: 10.1080/21642850.2018.1472602.

32. Cortina JM. What is coefficient alpha? An examination of theory and applications. J Appl Psychol. 1993;78(1):98.

33. Koo TK, Li MY. A Guideline of Selecting and Reporting Intraclass Correlation Coefficients for Reliability Research. J Chiropr Med. 2016;15(2):155-63. doi: 10.1016/j.jcm.2016.02.012.

34. Li C-H. Confirmatory factor analysis with ordinal data: Comparing robust maximum likelihood and diagonally weighted least squares. Behav Res Methods. 2016;48(3):936-49. doi: 10.3758/s13428-015-0619-7.

35. Hu Lt, Bentler PM. Cutoff criteria for fit indexes in covariance structure analysis: Conventional criteria versus new alternatives. Structural equation modeling: a multidisciplinary journal. 1999;6(1):1-55.

36. Council for International Organizations of Medical Sciences. International ethical guidelines for biomedical research involving human subjects. Bulletin of medical ethics. 2002;(182):17.

37. Labott SM, Johnson TP, Fendrich M, Feeny NC. Emotional risks to respondents in survey research: Some empirical evidence. Journal of Empirical Research on Human Research Ethics. 2013;8(4):53-66.

38. McClatchey IS, King S. The Impact of Death Education on Fear of Death and Death Anxiety Among Human Services Students. Omega (Westport). 2015;71(4):343-61. Epub 2015/12/17. doi: 10.1177/0030222815572606. PubMed PMID: 26665963.

39. Westerlund C, Tishelman C, Benkel I, Furst CJ, Molander U, Rasmussen BH, et al. Public awareness of palliative care in Sweden. Scand J Public Health. 2018;46(4):478-87. Epub 2018/01/06. doi: 10.1177/1403494817751329. PubMed PMID: 29301481; PubMed Central PMCID: PMCPMC5989249.

40. Arthur EK, Menon U, Browning K, Overcash J, Wills CE. Challenges of Cognitive Interviewing in Sensitive Health Topic Research. Nurs Res. 2021;70(5):376-82. Epub 2021/06/27. doi: 10.1097/NNR.0000000000000530. PubMed PMID: 34173378.

41. Terwee CB, Bot SDM, De Boer MR, Van Der Windt DAWM, Knol DL, Dekker J, et al. Quality criteria were proposed for measurement properties of health status questionnaires. J Clin Epidemiol. 2007;60(1):34-42. doi: 10.1016/j.jclinepi.2006.03.012.

42. Reeve BB, Fayers P. Applying item response theory modeling for evaluating questionnaire item and scale properties. Assessing quality of life in clinical trials: methods of practice. 2005;2:55-73.

43. Groeneveld EI, Cassel JB, Bausewein C, Csikós Á, Krajnik M, Ryan K, et al. Funding models in palliative care: Lessons from international experience. Palliat Med. 2017;31(4):296-305. doi: 10.1177/0269216316689015.

44. Lindqvist O, Tishelman C. Going public: reflections on developing the DöBra research program for health-promoting palliative care in Sweden. Prog Palliat Care. 2016;24(1):19-24. doi: 10.1080/09699260.2015.1103497. PubMed PMID: WOS:000376256700006.

45. Mohseni M, Lindström M. Social capital, trust in the health-care system and self-rated health: The role of access to health care in a population-based study. Soc Sci Med. 2007;64(7):1373-83. doi: 10.1016/j.socscimed.2006.11.023.

46. Nielsen JH, Lindvall J. Trust in government in Sweden and Denmark during the COVID-19 epidemic. West European Politics. 2021:1-25.

47. Statistics Sweden. Befolkningens utbildning 2020 [Educational attainment of the population 2020]. 2021.

48. Statistics Sweden. Befolkningsstatistik i sammandrag 1960–2020 [A summary of population statistics 1960-2020]. 2021.

49. Mercer AW, Kreuter F, Keeter S, Stuart EA. Theory and practice in nonprobability surveys: parallels between causal inference and survey inference. Public Opin Q. 2017;81(S1):250-71.

50. Valliant R. Comparing alternatives for estimation from nonprobability samples. Journal of Survey Statistics and Methodology. 2020;8(2):231-63.

51. Nolte S, Osborne RH, Dwinger S, Elsworth GR, Conrad ML, Rose M, et al. German translation, cultural adaptation, and validation of the Health Literacy Questionnaire (HLQ). PLoS One. 2017;12(2):e0172340. doi: 10.1371/journal.pone.0172340.

52. Han K, Colarelli SM, Weed NC. Methodological and statistical advances in the consideration of cultural diversity in assessment: A critical review of group classification and measurement invariance testing. Psychol Assess. 2019;31(12):1481.

53. Hayes B, Fabri AM, Coperchini M, Parkar R, Austin-Crowe Z. Health and death literacy and cultural diversity: insights from hospital-employed interpreters. BMJ Support Palliat Care. 2020;10(1):e8. Epub 2017/07/06. doi: 10.1136/bmjspcare-2016-001225. PubMed PMID: 28676498.

54. Collins A, Brown JEH, Mills J, Philip J. The impact of public health palliative care interventions on health system outcomes: A systematic review. Palliat Med. 2020. doi: 10.1177/0269216320981722.

55. Sallnow ES. Collective social capital: a study of new public health and end-of-life care. University of Edinburgh2018.

56. Albers G, Pereira S, Onwuteaka-Philipsen B, Deliens L, Pasman R, Van den Block L. Chapter 1. A public health perspective on palliative care for older people: an introduction. In: Van den Block L, Albers G, Pereira S, Onwuteaka-Philipsen B, Pasman R, Deliens L, editors. Palliative care for older people: A public health perspective. Oxford: Oxford University Press; 2015. p. 3-15.

57. Bone AE, Gomes B, Etkind SN, Verne J, Murtagh FEM, Evans CJ, et al. What is the impact of population ageing on the future provision of end-of-life care? Population-based projections of place of death. Palliat Med. 2018;32(2):329-36. Epub 2017/10/11. doi: 10.1177/0269216317734435. PubMed PMID: 29017018; PubMed Central PMCID: PMCPMC5788077.

58. SOU. God och nära vård: En reform för ett hållbart hälso-och sjukvårdssystem (SOU 2020:19) [Good and close care: A reform for a sustainable health care system]. Stockholm: Regeringskansliet, Stockholm, 2020.

59. Iida K, Ryan A, Hasson F, Payne S, McIlfatrick S. Palliative and end-of-life educational interventions for staff working in long-term care facilities: An integrative review of the literature. Int J Older People Nurs. 2021;16(1). doi: 10.1111/opn.12347.

60. Johansson T, Tishelman C, Cohen J, Eriksson LE, Goliath I. Continuums of change in a competence-building initiative addressing end-of-life communication in Swedish elder care. Qual Health Res. 2021;31(10):1904-17. doi: 10.1177/10497323211012986.

Supporting information

S1 File. Original English-language Death Literacy Index, with scale and sub-scale headings

S2 File. **Online survey, comprising the Swedish Death Literacy Index items and sociodemographic questions (in English).**

S3 File. Instructions for expert panel review

S4 Table. **Matrix with detailed examples of the revision process of two DLI-S items throughout instrument adaptation and validation.**

Notes: DLI-S items 4 and 19, which were found to be problematic in terms of clarity, relevance, and/or language, are presented in Swedish and English. Item revision is marked in bold, with reasoning presented in English.

S5 Table. Ratings of relevance and clarity and calculated overall content validity index (CVI).

Notes: *Positive rating=number of experts rating the item 3 or 4 on a 4-point scale. I-CVI calculated as (*n* of raters rating 3 or 4/total *n* of raters). Scale-level CVI was calculated using average proportion of I-CVI values.

S6 Table. Inter-item correlation matrix of DLI-S items.

**. Correlation is significant at the 0.01 level (2-tailed).
*. Correlation is significant at the 0.05 level (2-tailed).

Figure 1

Click here to access/download;Figure;Fig 1.pdf

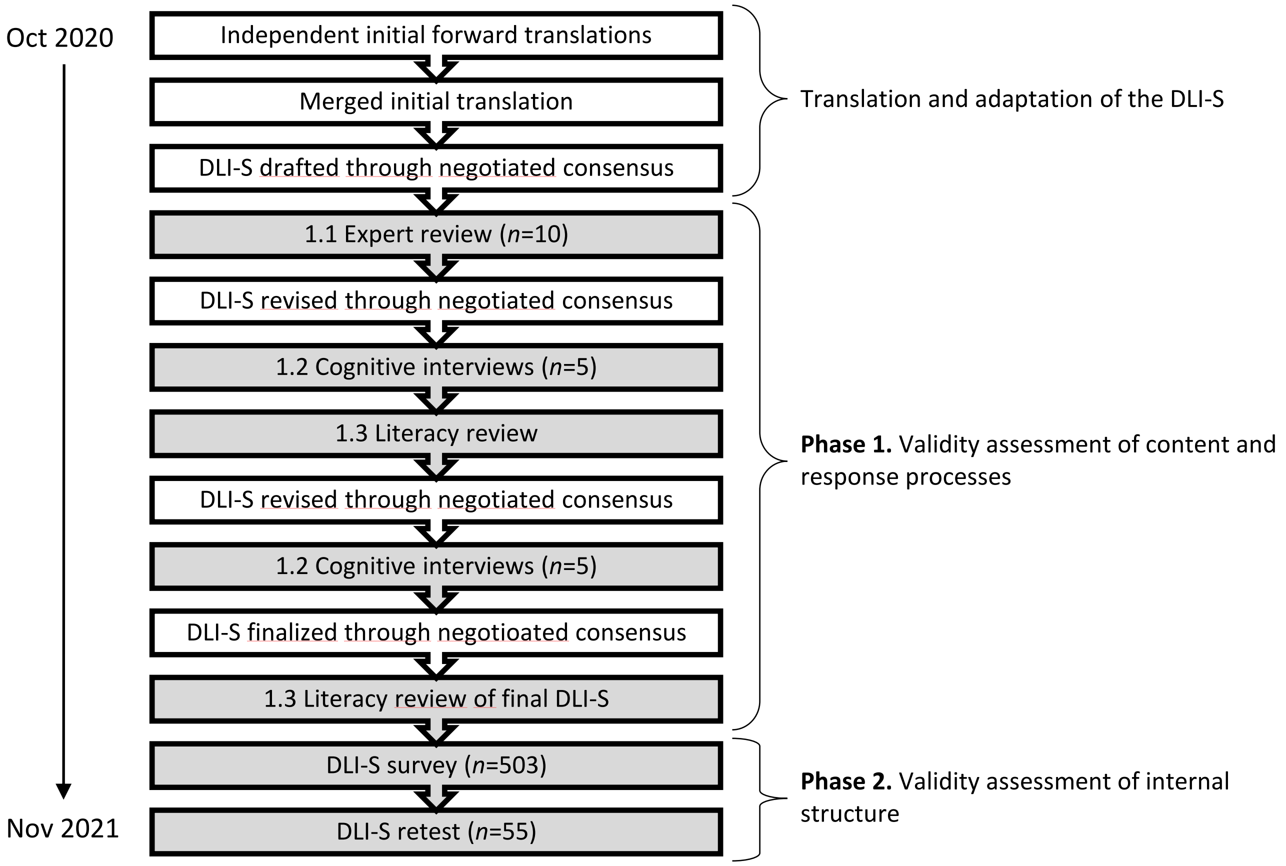

Figure 2

Click here to access/download;Figure;Fig 2.pdf

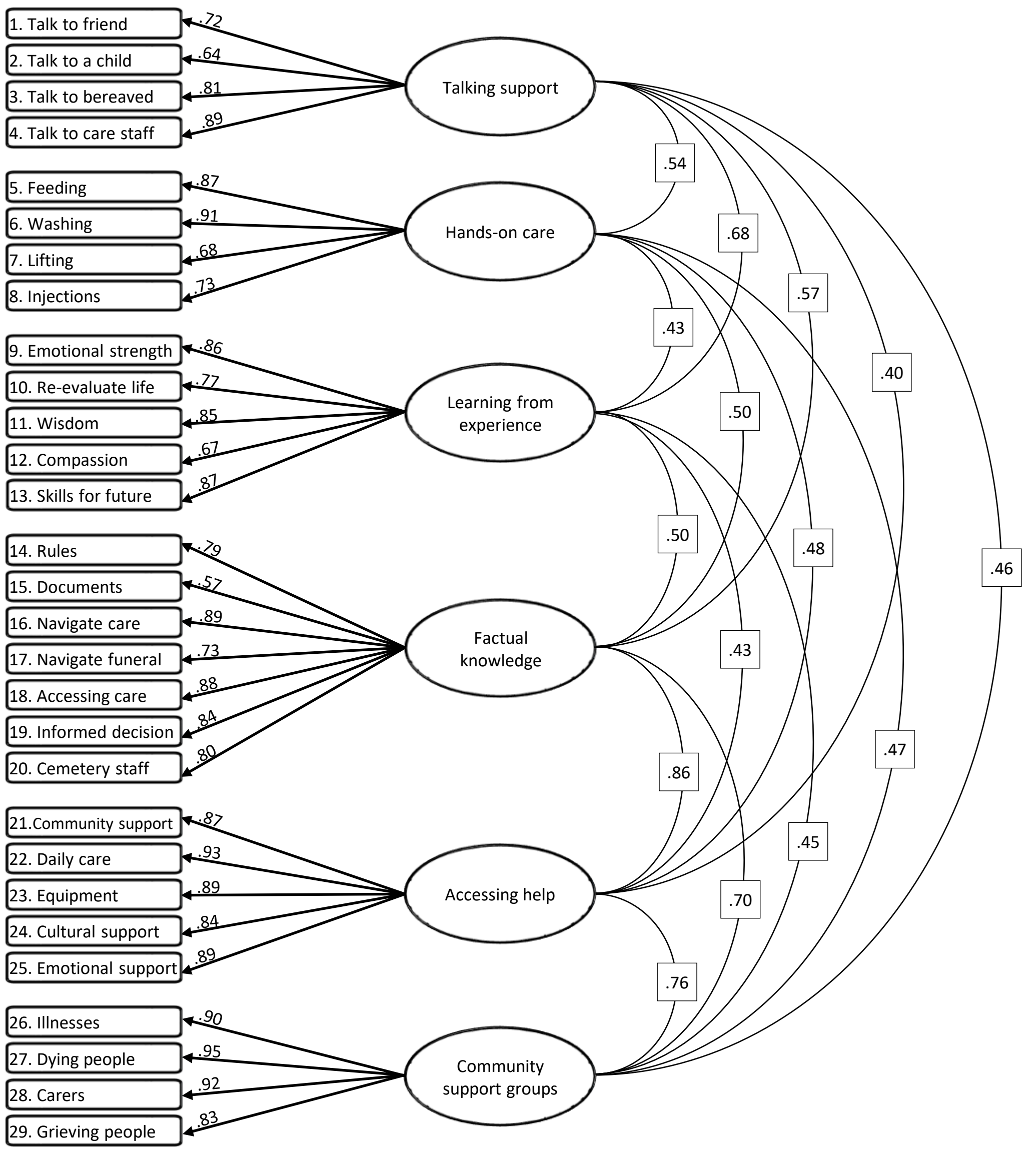

Click here to access/download
Supporting Information
S1 File.docx

Click here to access/download
Supporting Information
S2 File.docx

Click here to access/download
Supporting Information
S3 File.docx

Click here to access/download
Supporting Information
S4 Table.docx

Click here to access/download
Supporting Information
S5 Table.docx

Click here to access/download
Supporting Information
S6 Table.pdf
