# Supplementary material for: Validation of a culturally adapted Swedish-language version of the Death Literacy Index
Source: PLoS One. 2023 Nov 30;18(11):e0295141. doi: 10.1371/journal.pone.0295141 (PMC10688853; doi:10.1371/journal.pone.0295141)
Supplement: Attachment — Submitted filename: Response to reviewers.docx [file pone.0295141.s008.docx]

## Response to reviewers’ comments on article *Validation of a culturally adapted Swedish-language version of the Death Literacy Index (PONE-D-22-01686)*

| Reviewers’ comments | Author’s response to comments |
| --- | --- |
| **Editor** |  |
| No comments |  |
| **Reviewer 1** | We thank the reviewer for taking the time to read our submitted manuscript and for their helpful comments and suggestions. Changes in response to feedback have been carefully considered. Please see our specific responses below. |
| 1. The manuscript should be completed more. Reader need to know more about DL and DLI. | We thank the reviewer for pointing this out. While it should be noted that DL and the DLI has not yet been extensively studied, we have complemented the manuscript with more information about the theoretical construct and development of the measure in the introduction and aim sections (page 4, lines 56-67, added information in red):  *“The concept of DL was developed by an Australian research group after extensive qualitative research involving carers and networks involved in caring for a person dying at home.”* *Building on new public health approaches that highlight individual and community capacity-building and empowerment to handle issues of dying, death, and loss [3], DL is theorized to develop from engaging with EOL care and learning from those experiences. It is defined as “a set of knowledge and skills that make it possible to gain access to, understand, and act upon end-of-life and death care options” [4]. The death literacy index (DLI), an operationalization of DL, is a questionnaire developed from research in Australia about people’s experiences of EOL care [5]. The DLI evolved through an iterative mixed-methods approach to content development, refinement, and testing, and is intended to measure group levels of DL and evaluate* *EOL-related educational initiatives [6].*  We also made the following addition to provide more information about the DLI in the methods section (pages 5-6, lines 93-96):  *The proposed six-factor model in the original Australian DLI demonstrated satisfactory psychometric properties (CFI=0.955, TLI=0.95, RMSEA=0.049) indicating good model fit overall, and high reliability of the scales and sub-scales (Cronbach’s α ranging from 0.82 to 0.95) [2].* |
| 2. Materials and Methods: It is not complete because the process of Beaton et al. has not been completed. Why? | We recognise that we have not completed all steps set out in Beaton et al’s translation and adaptation process. We have also moved the sentence describing why we did not follow the step of back translation, so that it comes in direct connection to the reference. We have therefore made the following changes on (page 6, line 99-103):  “*After obtaining permission to translate the DLI from the original developers, co-authors RL and KN, the DLI-S development process was guided by recommendations for translation and cross-cultural adaptation [13, 14]. Back translation was not used in this study as it has been criticized for missing variation in linguistic meaning and cultural nuances [11, 15, 16].”* |
| 3. Materials and Methods: study design is not correct. | We are not sure exactly what the reviewer is critical of in this comment, nor why. Validation studies can be considered a study design in its own right, as they follow a standardized scientific approach (Arafat, 2016). We have added this reference to the description of the study design (page 5, line 77).  We argue that with consideration of the aim of this study (to develop, culturally adapt, and validate a Swedish-language DLI version with consideration to potential differences in the conceptualization and operationalization of death literacy), we have chosen an appropriate study design that follows the recommended key steps for translation, cross-cultural adaptation and validation (e.g., Behr & Shishido, 2016; Sousa et al. 2011).  We have updated the references in the Materials and methods section to demonstrate the appropriateness of the design based on best practice and quality criteria (Behr & Shishido, 2016; Sousa et al. 2011; Terwee et al. 2007). While we decided to exclude back translation, we provide justified reasons for doing so in the methods section (page 6, lines 102-103).  We have also made the following addition to justify our translation and validation procedure in the methods (page 7, lines 124-125):  *These correspond with identified quality criteria for assessing psychometric properties of measures [12]*.  A similar change has been made in the Strengths and limitations section (page 24, lines 455-457):  *Despite these limitations, the study complied with recommended practice for translation, cultural adaptation, and validation, applying a rigorous process to assess validity and reliability from numerous sources of evidence [12, 18, 19].*  Additional reasoning for the appropriateness of the study design is also provided in point 2 (above) and 4 (below). |
| 4. Usually Survey methods for harmonization of a scale or test are not valid. | We appreciate that the reviewer raises this concern. However, while the general purpose and procedure of harmonization aligns with the objectives for survey validation, a key difference is that harmonization is used for generating comparative multinational data using standardized means of collection and documentation (Wysmułek et al., 2022; Behr & Shishido, 2016).  Our aim was to develop and test the conceptual and functional relevance and validity of DLI items in a Swedish setting specifically, not for international use. In addition, use of the DLI is not primarily to compare death literacy between countries, but study relationships between variables that influence group-level death literacy and to identify need for, and evaluate impact of, education initiatives (Leonard et al., 2020, 2021).  We, and the original DLI developers acknowledge that the DLI items are influenced by several factors, e.g., language, culture and religion which affects death systems. The objective was therefore not to harmonize a cross-national, but rather explore the validity evidence in a country different from the original.  Furthermore, we acknowledge that the use of the term instrument in the article might erroneously lead the reader to think of a clinical instrument or test and have therefore changed this term to ‘questionnaire’ or ‘measure’ throughout the manuscript. |
| 5. How did you control inclusion and exclusion criteria for participants? | We thank the reviewer for pointing out that information about inclusion and exclusion criteria were missing for participants at two stages in the validation process. We have corrected this by specifying inclusion and exclusion criteria used in the following stages:  Cognitive interviews (page 8, lines 151-153): “*No explicit inclusion or exclusion criteria were used but we strove for variation in age, gender, educational level, and professional background*.”  Presumptive participants invited to the expert review panel and cognitive interviews were informed about the inclusion criteria. We did not control for either inclusion criteria beyond providing this information.  Survey panel (page 9, lines 169-170): “*The inclusion criteria were being eighteen years old or over and residing in Sweden. No explicit exclusion criteria were used and no EOL experience was required to participate.*”  The survey panel agency controlled the inclusion and exclusion criteria that had been determined for the survey participants (age 18 or over and resident in the country). Personal invitations were sent to eligible panel members, so self-selection was not possible. |
| 6. There are not inclusion and exclusion criteria in the text. | Please see our response to point 5 above. |
| 7. I didn't find psychometric parameters of the original scale, and authors didn't compare their data with it too. | We appreciate this remark and have added the following information about the model fit and reliability from the psychometric evaluation of the original DLI scale (pages 5-6, lines 93-96):  “*The proposed six-factor model in the original Australian DLI demonstrated satisfactory psychometric properties (CFI=0.955, TLI=0.95, RMSEA=0.049) indicating good model fit overall, and high reliability of the scales and sub-scales (Cronbach’s α ranging from 0.82 to 0.95).*” |
| 8. In discussion, authors don't have any comparison perspective for their data. It is the base for discussion section. | We agree that this would be useful to add to the discussion section. At the time of submission, there were no other validation studies published, but we have added references for three DLI validation articles that have subsequently been published and considered our findings in relation to these at the following places in the manuscript:  1 (page 20-21, lines 374-378): “*The results show evidence of cross-cultural validity of the DLI and confirm fit of the proposed six-factor model of DL. This supports previous findings, indicating that the theoretical model operationalized in the DLI is stable across countries despite differences in language, culture, and organisation of institutions that all might influence local or regional death systems.”*  2 (page 22, lines 417-420): “*This finding also points to differences between the Swedish and Australian context that appear as variation in item difficulty for these items [47]. This distinctiveness of the Swedish context is further emphasized given that Che et al. found the Community capacity scale to function well in China [9].*  3 (page 25, lines 477-479): *Nevertheless, more research is needed to better understand the DL construct, particularly across cultures, and to determine whether the DLI is appropriate to capture individual and community capacity for EOL in a comparable manner.* |
| **Reviewer 2** | We thank the reviewer for taking the time to read our submitted manuscript and for their helpful comments and suggestions. Changes in response to feedback have been carefully considered. Please see our specific responses below. |
| 1. Please provide the content validity index and fit indexes value(s) in the abstract. | The indices have been added to the abstract (page 2, lines 39 and 41). |
| 2. Can you provide one or two sentences about the validity of the original DLI | We agree with the reviewer more background about the original DLI would be helpful. We have therefore added information about the theoretical construct of DL and development of the DLI in the introduction and aim section (page 4, lines 56-67):  *“The concept of DL was developed by an Australian research group after extensive qualitative research involving carers and networks involved in caring for a person dying at home.”* *Building on new public health approaches that highlight individual and community capacity-building and empowerment to handle issues of dying, death, and loss [3], DL is theorized to develop from engaging with EOL care and learning from those experiences. It is defined as “a set of knowledge and skills that make it possible to gain access to, understand, and act upon end-of-life and death care options” [4]. The death literacy index (DLI), an operationalization of DL, is a questionnaire developed from research in Australia about people’s experiences of EOL care [5]. The DLI evolved through an iterative mixed-methods approach to content development, refinement, and testing, and is intended to measure group levels of DL and evaluate EOL-related educational initiatives [6].*  We also made the following addition to provide more information about the DLI in the methods section (pages 5-6, lines 93-96): *The proposed six-factor model in the original DLI demonstrated satisfactory psychometric properties (CFI=0.955, TLI=0.95, RMSEA=0.049) indicating good model fit overall, and high reliability of the scales and sub-scales (Cronbach’s α ranging from 0.82 to 0.95) [2]*. |
| 3. Is it possible to merge all phases and sub-phases' data analysis together? | We thank the reviewer for this suggestion. All phases and steps in data collection and analysis were presented together in an earlier version of the manuscript. We did, however, receive feedback that this was not optimal given the iterative procedure of translation, adaptation, and validation, where the results of each step influenced how the next was carried out. If the editor and reviewer find it beneficial to the article, however, we are willing to re-organize and merge the phases of data collection and data analysis in the (see pages 7-12 in the methods section). We have also removed the reference to Phase 1 and 2 in Figure 1. |
| 4. It will be good to perform exploratory factor analysis in addition to confirmatory factor analysis. This will tell the reader the best model supported by the current data | We appreciate that the reviewer highlights this question, as it is a methodologically important issue to raise that was discussed with our statistical advisors as we planned and carried out this study. Since there was already a hypothesised model that had been identified and tested during the development of the original DLI, a confirmatory factor analysis (CFA) was considered the appropriate option for the psychometric evaluation of the DLI-S (Knekta et al., 2019). Since we have conducted a CFA on the sample, which fit the data well, it has been suggested questionable to perform an exploratory factor analysis (EFA) on the same data set as it will likely replicate a similar factor structure to the CFA (Bandalos & Finney, 2010). |
| 5. Can you move 'scale descriptives' up? i.e. before 'reliability' results. | This section has been moved as requested (page 19, lines 334-338). |
| 6. Can you change the sub-title 'Methodological discussion' to 'strength and limitation'? | The section has been re-named ‘Strengths and limitations’ (page 23, line 439). |
