# Supplementary material for: Validation of a culturally adapted Swedish-language version of the Death Literacy Index
Source: PLoS One. 2023 Nov 30;18(11):e0295141. doi: 10.1371/journal.pone.0295141 (PMC10688853; doi:10.1371/journal.pone.0295141)
Supplement: Attachment — Submitted filename: Response to reviewers_r2.docx [file pone.0295141.s009.docx]

## Response to reviewers’ comments on article *Validation of a culturally adapted Swedish-language version of the Death Literacy Index (PONE-D-22-01686R2)*

| Reviewers’ comments | Author’s response to comments |
| --- | --- |
| **Reviewer #1** | We thank the reviewer for reading our resubmitted manuscript and for their helpful feedback. Changes in response to feedback have been carefully considered. Please see our specific responses below with additions to the manuscript text added information in red. References to page and line numbers relate to the manuscript with track changes. |
| **Introduction**  Although you have mentioned about other language versions of the scale, but there are not any data about them. They are necessary. | We agree that this is necessary data and have now added data for reliability and model fit from the other language versions. To ensure coherence of the manuscript text we present this information in the section about the Death Literacy Index in the beginning of the methods section (p. 6, lines 100-107):  *The proposed six-factor model in the original English-language DLI demonstrated satisfactory psychometric properties in Australia indicating good model fit overall (CFI=0.955, TLI=0.95, RMSEA=0.049), and high reliability of the scales and sub-scales (Cronbach’s α ranging 0.82 to 0.95) [2], and the United Kingdom (CFI=0.94, RMSEA=0.07, SRMR=0.07; Cronbach’s α for sub-scales ranging 0.79 to 0.90) [7]. Moreover, translated DLI versions have demonstrated acceptable reliability and validity in Turkey (total CVI=0.91; CFI=0.93, RMSEA = 0.053; Cronbach’s α ranging 0.68-0.90) [8] and China (CFI=0.956, RMSEA=0.055, SRMR=0.051, Cronbach’s α for sub-scales ranging 0.76–0.97) [9].* |
| **Methods**  Survey panel characteristics are not enough. It needs to be explained more. | Since we used stratified sampling based on 64 cells (2 gender x 4 age groups x 8 NUTS2 regions in Sweden according to National Statistics Sweden population data), the survey panel composition for these characteristics is not relevant for the process of sample selection. Moreover, the survey panel characteristics are less important in this study, since our data should only be seen in terms of validation of the DLI, as we are not making any statistical inferences to the general population.  Nevertheless, we have added information about overall characteristics of the agency’s existing survey panel, e.g., that all members are aged 15 or over and Swedish residents (p. 9, lines 176-178):  *Data was collected from September–November 2021 through an online survey administered by an external data collection agency with a pre-existing panel of ca 100,000 Swedish residents,* *aged 15 or over, willing to partake in surveys on various topics for both market and scientific research.*  We have also clarified that our sample was drawn from an existing survey panel on page 9, lines 183-185:  *Survey invitations were sent to a quota sample (n=2991) in the agency’s existing panel, stratified by gender, age, and geographical region.*  Differences between our sample and the general Swedish population in characteristics that were not part of the stratified sampling (country of origin and education level) are also addressed in the strengths and limitations section in the discussion (page 24, lines 465-471). |
| The method of translation and adaptation should be more precisely. Authors have pointed to multi-step mixed methods approach, but without any explanation about it. | The steps are outlined in Figure 1, but we understand that the reader might want this information in the manuscript text as well. We have therefore described what the steps in the multi-step process entailed in the methods section (page 5, lines 79-85):  *This instrument validation study [10] used a multi-step approach combining qualitative and quantitative methods [11] to translate and culturally adapt the Death Literacy Index (DLI) into Swedish and to assess the validity of the resulting DLI-S [12]. The multi-step process comprised: forward translation and adaptation by initial translators; translation revision through negotiated consensus in the research team; review by a panel of experts; pre-testing using cognitive interviews; literacy reviews by an external consultant; and psychometric evaluation using online testing of the DLI-S with a survey panel (see Fig 1).* |
| **Results**  Authors should explain why they didn't do exploratory factor analysis. | We appreciate that the reviewer highlights this, as it is a methodologically important issue that was discussed with our statistical advisors when we planned and conducted this study. We have added information about why an EFA was not performed in the methods section (p. 13, lines 270-273):  *Since a hypothesized model had been identified and tested during the development of the original DLI, a CFA was considered more appropriate to assess the fitness of the DLI factor structure than an exploratory factor analysis [41, 42].* |
| **Discussion**  There isn't any comparison between results of the present study and other language versions. | We acknowledge that we can situate our results more in relation to other language-versions. We have therefore added more comparison of the validity of the DLI and the function of its scales between our study and prior validation studies throughout the discussion:  p. 21, lines 392-393: *This supports other recent validation studies [7-9] as well as the potential of the DLI to be used to measure DL internationally.*  p. 22, lines 395-396: *Both I-CVI and S-CVI ratings exceed the recommended minimum set out by Polit et al. [30], suggesting support for DLI-S’ validity in terms of item clarity and relevance for the DL construct, which aligns with Turkish results.*  p. 22, lines 423-424: *The DLI-S was found to have satisfactory psychometric properties, with support for validity evidence based on internal structure, which corresponds to previous findings in validations of other language-versions of the DLI [2,7-9].*  p. 22, lines 436-437: *The distinctiveness of the Swedish context is further emphasized as the Community capacity scale functions well in China and Turkey [8, 9].*  p. 23, lines 444-447: *Further studies are thus warranted to explore the performance of a shorter DLI-S version in the Swedish context and to investigate if a five-factor model constitutes a better fit for the DLI-S and the extent to which this might be relevant in other contexts. The Turkish and Chinese DLI validations similarly suggest that correlations between these two are the highest of the DLI scales, though not exceeding the cut-off off 0.85. This could indicate that knowledge about formal and informal systems and processes for care provision and support are not easily distinguished.*  p. 23, lines 450-453: *In sum, the validity evidence for internal structure show that the DLI-S performs well psychometrically, although the comparatively worse performance of the Community capacity sub-scales may indicate difference in function or meaning in Sweden compared to Australia, Turkey, China, and the United Kingdom. This finding could be an accurate reflection of differences in context, particularly in how care systems are organized and people’s expectations of and interactions with them.*  p. 24, lines 480-482: *In comparison to earlier DLI validations, we investigate the factor structure of DL in more detail, adding to the understanding of the fit between theoretical and factor model.*  Our previous overarching reference to psychometric evaluations of other DLI versions has been moved to the conclusion. |
| **Reviewer #2** |  |
| **Abstract discrepancies**  The manuscript has improved. There are two discrepancies noticed in the abstract, text body, table 4 and figure 2 which have not been attended to.  The sub scale alpha ranges between 0.81 and 0.92. Please correct it in the abstract and results sections (0.81 - 0.93).  The correlations between factors range between 0.40 and 0.86 in figure 2. Please correct it under results section (0.43 - 0.86). | We thank the reviewer for reading our resubmitted manuscript and for their helpful feedback. We have corrected the identified typos: the Cronbach’s alpha range for the sub-scales in the abstract (p. 2, line 43) and results (p. 19, line 354), and the factor correlation range in the results (p. 19, line 362).  We thank the reviewer for reading our resubmitted manuscript and for their helpful feedback. We have corrected the identified typos:  - the Cronbach’s alpha range for the sub-scales in:  - the abstract (p. 2, line 43): *High internal consistency reliability was demonstrated for the overall scale (Cronbach’s α=0.94) and each sub-scale (α 0.81–0.92)*  - the results (p. 19, line 354): *The DLI-S demonstrated high internal consistency reliability, Cronbach’s α=0.94 for the overall scale and between 0.81–0.92 for the sub-scales.*  - The factor correlation range in the results (p. 19, line 362): *Factor loadings in this model were generally high, ranging between 0.57–0.95, and correlations between factors were moderate to strong, between 0.40–0.86 (Fig 2).* |
